# Epidemiology of 40 blood biomarkers of one-carbon metabolism, vitamin status, inflammation, and renal and endothelial function among cancer-free older adults

**DOI:** 10.1038/s41598-021-93214-8

**Published:** 2021-07-05

**Authors:** Hana Zahed, Mattias Johansson, Per M. Ueland, Øivind Midttun, Roger L. Milne, Graham G. Giles, Jonas Manjer, Malte Sandsveden, Arnulf Langhammer, Elin Pettersen Sørgjerd, Kjell Grankvist, Mikael Johansson, Neal D. Freedman, Wen-Yi Huang, Chu Chen, Ross Prentice, Victoria L. Stevens, Ying Wang, Loic Le Marchand, Lynne R. Wilkens, Stephanie J. Weinstein, Demetrius Albanes, Qiuyin Cai, William J. Blot, Alan A. Arslan, Anne Zeleniuch-Jacquotte, Xiao-Ou Shu, Wei Zheng, Jian-Min Yuan, Woon-Puay Koh, Kala Visvanathan, Howard D. Sesso, Xuehong Zhang, J. Michael Gaziano, Anouar Fanidi, David Muller, Paul Brennan, Florence Guida, Hilary A. Robbins

**Affiliations:** 1 Genomic Epidemiology Branch, International Agency for Research on Cancer, 150 cours Albert Thomas, 69008 Lyon, France; 2 Department of Clinical Science, University of Bergen, Bergen, Norway; 3 Bevital AS, Bergen, Norway; 4 Cancer Epidemiology Division, Cancer Council Victoria, Melbourne, Australia; 5 Centre for Epidemiology and Biostatistics, School of Population and Global Health, The University of Melbourne, Melbourne, Australia; 6 Precision Medicine, School of Clinical Sciences at Monash Health, Monash University, Melbourne, Australia; 7 Department of Surgery, Skane University Hospital, Malmö, Sweden; 8 Lund University, Malmö, Sweden; 9 Department of Clinical Sciences Malmo, Lund University, Malmö, Sweden; 10 Department of Public Health and Nursing, Hunt Research Centre, Norwegian University of Science and Technology, Levanger, Norway; 11 Levanger Hospital, Nord-Trøndelag Hospital Trust, Levanger, Norway; 12 Department of Public Health and Nursing, NTNU, Hunt Research Centre, Norwegian University of Science and Technology, Levanger, Norway; 13 Department of Endocrinology, St. Olavs Hospital, Trondheim University Hospital, Levanger, Norway; 14 Department of Medical Biosciences, Umea University, Umeå, Sweden; 15 Department of Radiation Sciences, Oncology, Umea University, Umeå, Sweden; 16 Metabolic Epidemiology Branch, Division of Cancer Epidemiology and Genetics, National Cancer Institute, Bethesda, MD USA; 17 Public Health Sciences Division, Fred Hutchinson Cancer Research Center, Seattle, USA; 18 American Cancer Society, Atlanta, USA; 19 University of Hawai’i Cancer Center, University of Hawaiʻi at Mānoa, Honolulu, USA; 20 Vanderbilt University Medical Center, Nashville, USA; 21 Department of Obstetrics and Gynecology, NYU Langone Health, New York, NY USA; 22 Department of Population Health, NYU Langone Health, New York, NY USA; 23 Perlmutter Comprehensive Cancer Center, NYU Langone Health, New York, NY USA; 24 University of Pittsburgh Medical Center, Pittsburgh, USA; 25 Duke - NUS Medical School, Singapore, Singapore; 26 Johns Hopkins Institute for Clinical and Translational Research, Baltimore, USA; 27 Brigham and Women’s Hospital, Harvard Medical School, Boston, USA; 28 Harvard T.H. Chan School of Public Health, Boston, USA; 29 Brigham and Women’s Hospital, Boston, USA; 30 Université Claude Bernard Lyon 1, Lyon, France; 31 Imperial College London School of Public Health, London, UK

**Keywords:** Biomarkers, Epidemiology

## Abstract

Imbalances of blood biomarkers are associated with disease, and biomarkers may also vary non-pathologically across population groups. We described variation in concentrations of biomarkers of one-carbon metabolism, vitamin status, inflammation including tryptophan metabolism, and endothelial and renal function among cancer-free older adults. We analyzed 5167 cancer-free controls aged 40–80 years from 20 cohorts in the Lung Cancer Cohort Consortium (LC3). Centralized biochemical analyses of 40 biomarkers in plasma or serum were performed. We fit multivariable linear mixed effects models to quantify variation in standardized biomarker log-concentrations across four factors: age, sex, smoking status, and body mass index (BMI). Differences in most biomarkers across most factors were small, with 93% (186/200) of analyses showing an estimated difference lower than 0.25 standard-deviations, although most were statistically significant due to large sample size. The largest difference was for creatinine by sex, which was − 0.91 standard-deviations lower in women than men (95%CI − 0.98; − 0.84). The largest difference by age was for total cysteine (0.40 standard-deviation increase per 10-year increase, 95%CI 0.36; 0.43), and by BMI was for C-reactive protein (0.38 standard-deviation increase per 5-kg/m^2^ increase, 95%CI 0.34; 0.41). For 31 of 40 markers, the mean difference between current and never smokers was larger than between former and never smokers. A statistically significant (*p* < 0.05) association with time since smoking cessation was observed for 8 markers, including C-reactive protein, kynurenine, choline, and total homocysteine. We conclude that most blood biomarkers show small variations across demographic characteristics. Patterns by smoking status point to normalization of multiple physiological processes after smoking cessation.

## Introduction

Imbalances of blood biomarkers are associated with several diseases. For example, perturbations in one-carbon metabolism may lead to cardiovascular disease^1^, cancer^2–4^, and other diseases^5, 6^. Deficiencies of vitamins A, D and E are associated with increased risk of type II diabetes and multiple immune system disorders^7^. Accordingly, measurements of blood biomarkers have been studied extensively in relation to illness^8–13^, but their variation among healthy individuals is less commonly described.

The Lung Cancer Cohort Consortium (LC3) was initially established to provide evidence on the potential importance of biomarkers of one-carbon metabolism in lung cancer etiology among 20 prospective cohorts. The project measured markers of B-vitamins and fat-soluble vitamins, functional biomarkers such as one-carbon metabolites, inflammation markers including tryptophan metabolites of the kynurenine pathway, and markers of renal and endothelial function. Previous analyses of the LC3 primarily assessed the relationship of these markers with lung cancer risk^8–13^, while one study analyzed control participants to describe the variation in biomarkers across geographical regions^14^. Lung cancer risk was found to be positively associated with levels of vitamin B12^9^, vitamin B6^13^ and components of tryptophan metabolism^15^. Among cancer-free control participants, analyses by geography showed higher levels of vitamins in the USA due to widespread vitamin supplementation and food-fortification practices, while one-carbon and tryptophan metabolites were highest in Asian populations^14^.

Beyond patterns by geography, circulating levels of biomarkers can also vary within healthy populations across demographic and behavioral factors such as age^16, 17^, smoking history^18, 19^, and sex^20^. The large and diverse LC3 dataset offers an important opportunity to comprehensively describe biomarker variation among cancer-free older adults, but the prior analysis of control participants focused only on geographical variation. Here, we analyzed the LC3 control participants to describe variation in biomarkers of one-carbon metabolism, vitamin status, inflammation including tryptophan metabolites of the kynurenine pathway, and renal and endothelial function. We examined key demographic and behavioral factors, specifically age, sex, smoking status, and body mass index (BMI).

## Methods

### Study sample

We analyzed data from 20 cohorts in the Lung Cancer Cohort Consortium (LC3), including 11 US cohorts, 4 Nordic cohorts, 4 Asian cohorts, and 1 Australian cohort (see Table 1 footnote for details)^8, 11–14^. In each cohort, biomarkers were measured in plasma or serum samples from lung cancer cases and matched controls. Here, our aim was to describe patterns of blood biomarker status in cancer-free older adults, so we restricted analysis to the matched controls. These controls were matched individually to the cases by sex, date of blood collection, date of birth, smoking status in 5 categories (never smokers, former smokers with < 10 or ≥ 10 quit-years, and current smokers with < 15 or ≥ 15 cigarettes per day), and (for US cohorts only) race/ethnicity. All cohort participants provided written, informed consent.

**Table 1:** Descriptive characteristics of 5167 control subjects in the Lung Cancer Cohort Consortium.

Characteristic	N (%)
**Geographical region**	
USA	2259 (43.7%)
Nordic	803 (15.6%)
Asian	1747 (33.8%)
Australian	358 (6.9%)
**Age, years**	
49 or younger	473 (9.1%)
50 to 59	1448 (28.0%)
60 to 69	2324 (45.0%)
70 to 80	922 (17.9%)
**Sex**	
Male	2810 (54.4%)
Female	2357 (45.6%)
**Body mass index**	
Less than 18.5	121 (2.3%)
18.5 to < 25	2608 (50.5%)
25 to < 30	1802 (34.9%)
30 or higher	636 (12.3%)
**Smoking status**	
Never	1264 (24.5%)
Former	1453 (28.1%)
Current	2450 (47.4%)
**Race/ethnicity**	
White	2947 (57.0%)
Black	247 (4.8%)
Asian	1812 (35.1%)
Other	161 (3.1%)

The study population includes cancer-free control subjects from the Lung Cancer Cohort Consortium, which comprises 20 cohorts from the USA (Campaign Against Cancer and Stroke and Campaign Against Cancer and Heart Disease (CLUE, N = 171), American Cancer Society Cancer Prevention Study-II Nutrition Cohort (CPS-II, N = 179), Health Professionals Follow-Up Study (HPFS, N = 130), Multiethnic Cohort (MEC, N = 148), Nurses’ Health Study (NHS, N = 328), New York University Women’s Health Study (NYUWHS, N = 167), Physicians’ Health Study (PHS, N = 76), Prostate Lung Colorectal and Ovarian Cancer Screening Trial (PLCO, N = 440), Southern Community Cohort Study (SCCS, N = 209), Women’s Health Initiative (WHI, N = 228), Women’s Health Study (WHS, N = 183)), Nordic countries (Alpha-Tocopherol, Beta-Carotene Cancer Prevention Study (ATBC, N = 200), Trøndelag Health Study (HUNT, N = 174), Northern Sweden Health and Disease Study Cohort (NSHDS, N = 230), Malmö Diet and Cancer Study (MDCS, N = 199)), Asia (Shanghai Men’s Health Study (SMHS, N = 419), Shanghai Women’s Health Study (SWHS,N = 417), Shanghai Cohort Study (SCS, N = 502), Singapore Chinese Health Study (SCHS, N = 409)), and Australia (Melbourne Collaborative Cohort Study (MCCS, N = 358)). Details regarding the data from individual cohorts have been published previously^7^.

Before exclusions, numbers of control participants in each cohort ranged from 81 (Physicians’ Health Study) to 513 (Shanghai Cohort Study). Across the consortium, samples were collected between 1974 and 2010. Some cohorts included almost exclusively current and former smokers (for example the Southern Community Cohort Study included 73% current and 20% former smokers) while in others, never smokers were intentionally over-sampled (Women’s Health Initiative, 100% never smokers). Blood samples were drawn at baseline when participants were required to be free of any cancer (except non-melanoma skin cancer). Controls were required to be free of cancer at the time of diagnosis of the matched case, and the median time between blood collection and diagnosis of the index cancer case was 7.0 years (interquartile range 3.3 to 11.0 years).

Individuals with extreme values of covariates (e.g. continuous covariates such as BMI and age) can have high influence on estimated coefficients. To avoid this, we excluded individuals with BMI outside the range of 17–40 kg/m^2^ (N = 138) and subsequently excluded individuals with age at blood draw outside the range of 40–80 years (N = 84). Therefore, from 5389 individuals, 222 were excluded and the final study sample included 5167 participants.

### Biomarker measurements

Biomarker measurements in serum or plasma were performed at the BEVITAL laboratory in Bergen, Norway using mass spectrometry based and microbiological methods as described in Supplementary Table 1. Samples in each cohort were divided into batches of approximately 86 samples. All samples in a single batch were derived from the same cohort and a batch adjustment was included in analyses (see below). Between-batch coefficients of variation (CVs) ranged from 2 to 15%, with the exception of methionine sulfoxide which had a CV of 29%^14^.

We divided the 40 biomarkers into 4 categories: (1) one-carbon metabolism markers (methionine, methionine-sulfoxide, total homocysteine, cystathionine, total cysteine, serine, glycine, choline, betaine, dimethylglycine, and sarcosine), (2) vitamins including B-vitamins (pyridoxal 5′-phosphate (PLP), pyridoxal (PL), 4-pyridoxic acid (PA), riboflavin, nicotinamide, folate and cobalamin and methylmalonic acid), and fat-soluble vitamins (vitamin A measured as all trans-retinol metabolites, 25OH-vitamin D, alpha-tocopherol, gamma-tocopherol), (3) tryptophan metabolites of the kynurenine pathway (tryptophan, kynurenine, kynurenic acid, anthranilic acid, 3-hydroxykynurenine, xanthurenic acid, 3-hydroxyanthranilic acid, quinolinic acid) and inflammation markers (C-reactive protein (CRP), total neopterin, kynurenine/tryptophan ratio (KTR), Par index $\left( {\frac{{PA}}{{PLP + PL}}} \right)$), and (4) markers of endothelial or renal function (arginine, homoarginine, asymmetric dimethylarginine (ADMA), symmetric dimethylarginine (SDMA), and creatinine).

Blood samples were stored for different lengths of time in each cohort, but there was also substantial variation in storage time within cohorts (e.g. NSHDS samples were stored for between 0 and 21 years). A storage time adjustment was included in the analysis (see below). Information on blood collection dates by cohort is provided in Supplementary Table 2.

### Statistical analysis

We calculated the PAr index, which serves as a measure of vitamin B6 catabolism, as the ratio of pyridoxic acid to the sum of pyridoxal and PLP concentrations^13^. We calculated the kynurenine-tryptophan ratio, which serves as a measure of cellular immune activation, by dividing kynurenine concentrations by tryptophan concentrations^15^. Concentrations of total vitamin D were calculated by adding the concentrations of 25(OH)D2 to seasonally adjusted concentrations of 25(OH)D3. Because 25(OH)D3 is strongly affected by exposure to UVB radiation, we performed seasonal adjustments using Bayesian hierarchical regression models^21^. Seasonally adjusted 25(OH)D3 was estimated by adding or subtracting the estimated deviation from the mean concentration according to the day of blood draw. We applied a logarithmic base-2 transformation to all biomarker measurements and ratios. We then standardized all values by centering on the mean and dividing by the standard deviation.

To compare biomarker values across our variables of interest (age, sex, smoking status, and BMI), we used multivariable linear mixed effects models. We fit one model for each biomarker with the log-transformed, standardized biomarker value as the outcome. Each model included fixed effects for age (continuous), sex (female vs. male), smoking status (never, former, current), BMI (continuous), and random effects for batch. Models were adjusted using fixed effects for cohort, race/ethnicity, and freezer storage time. Using this approach, each model coefficient (noted as β) represents the mean change in the log-biomarker, in standard-deviation units, associated with a 1-unit change in the independent variable. For age and BMI, we re-scaled these coefficients to represent the effect of a 10-year increase in age and a 5-unit increase in BMI. While we adjusted for race/ethnicity, we did not report these results because racial/ethnic differences in our dataset mostly occur across geographical regions, and this was the subject of a prior manuscript^14^.

Finally, we quantified proportions of vitamin deficiency for riboflavin, PLP, folate, cobalamin, vitamin A, alpha-tocopherol and vitamin D across demographic categories, using previously established cutpoints for vitamin deficiency^14^**.** To investigate associations with time since quitting smoking, we restricted to cohorts that provided data on quitting time among former smokers, then assessed whether the difference in biomarker measurements between former smokers and never smokers attenuated as time since quitting increased.

We performed multiple sensitivity analyses. For the modeling approach, we compared the primary analysis results to results from three different changes to the model. The first excluded the fixed effect for cohort, the second excluded the random effect for batch, and the third excluded the fixed effect for storage time (Supplementary Methods). We also assessed the effect of excluding single-sex cohorts on the coefficients for sex, the effect of excluding outlying values of the log-biomarkers on all results, and the assumption of linear relationships between each biomarker and age/BMI.

All analyses were done in R versions 3.5.3, 3.6, and 4.0. Mixed effects models were fit using the lme4 package.

### Ethical approval

The protocol of the Lung Cancer Cohort Consortium was approved by the Ethics Committee of the International Agency for Research on Cancer (Project number 11-13). The recruitment, data collection, and follow-up of the participating cohorts was approved by local institutional review boards. This study involved no additional contact or intervention with participants. This research was performed in accordance with the Declaration of Helsinki.

## Results

Baseline characteristics of the study sample are presented in Table 1, and stratified characteristics by cohort are presented in Supplementary Table 3. Geographically, 43.7% of the population was from the USA, with smaller proportions from Asia (33.8%), Europe (15.6%), and Australia (6.9%). Of all participants, 54% were male, and 57% were of white race/ethnicity. The mean age was 61.7 years with a standard deviation of 8.3. The majority of the sample was comprised of current smokers (47.4%) and former smokers (28.1%). Storage duration of serum/plasma samples had a modest impact on log biomarker concentrations. Among all biomarkers, the median change in log-concentration was 0.01 standard deviations per year stored, and the maximum change was 0.04 standard deviations per year stored (Supplementary Table 4).

**Figure 1:**
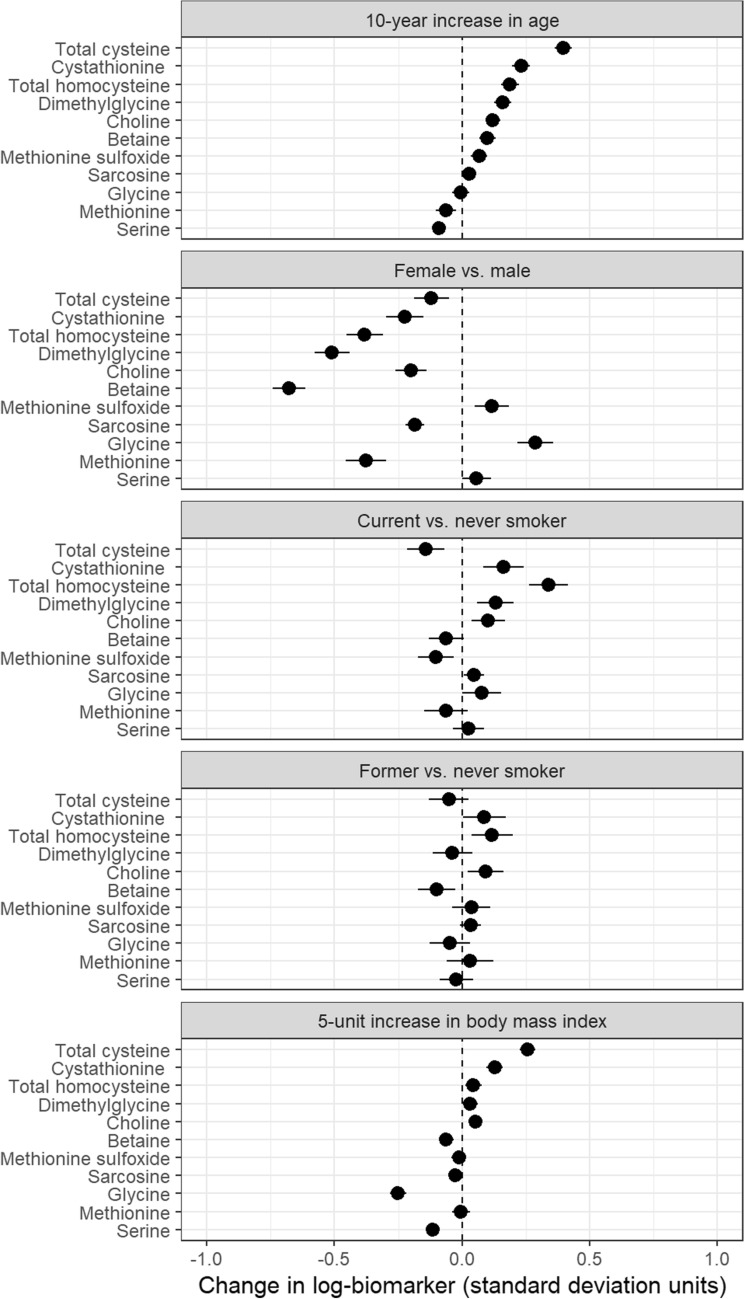
Differences in biomarkers of one-carbon metabolism by age, sex, smoking status, and BMI, in units of log-biomarker standard deviations, among 5167 control subjects in the Lung Cancer Cohort Consortium. Biomarkers are listed in order of the age coefficient.

**Figure 2:**
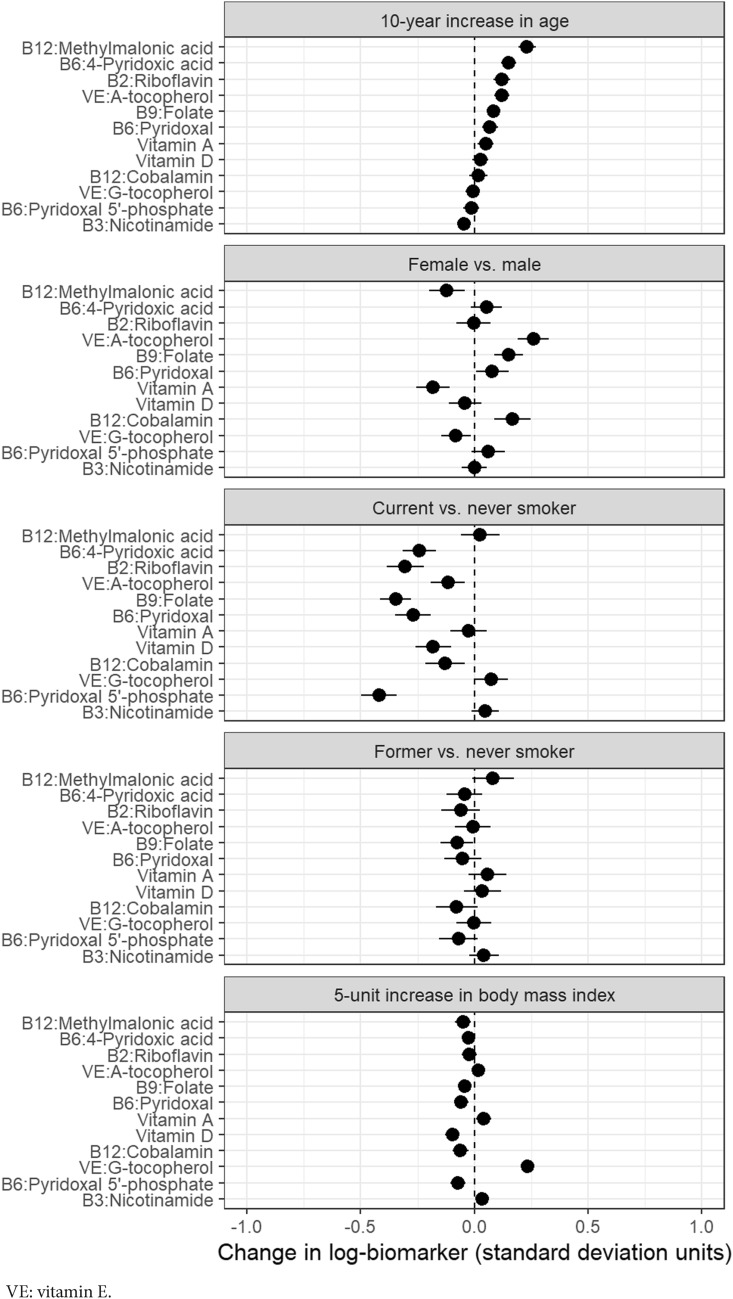
Differences in biomarkers of vitamin status by age, sex, smoking status, and BMI, in units of log-biomarker standard deviations, among 5167 control subjects in the Lung Cancer Cohort Consortium. Biomarkers are listed in order of the age coefficient.

**Figure 3:**
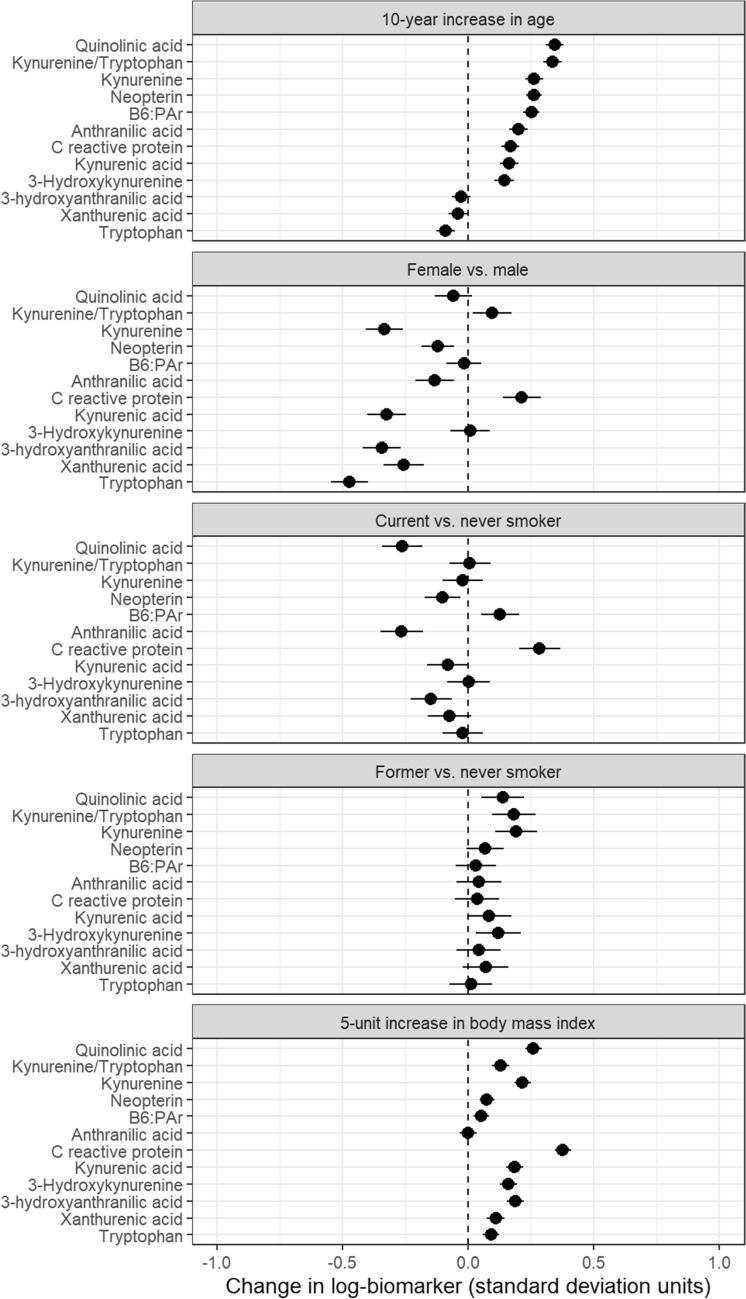
Differences in biomarkers of the kynurenine pathway and inflammation by age, sex, smoking status, and BMI in units of log-biomarker standard deviations, among 5167 control subjects in the Lung Cancer Cohort Consortium. Biomarkers are listed in order of the age coefficient.

**Figure 4:**
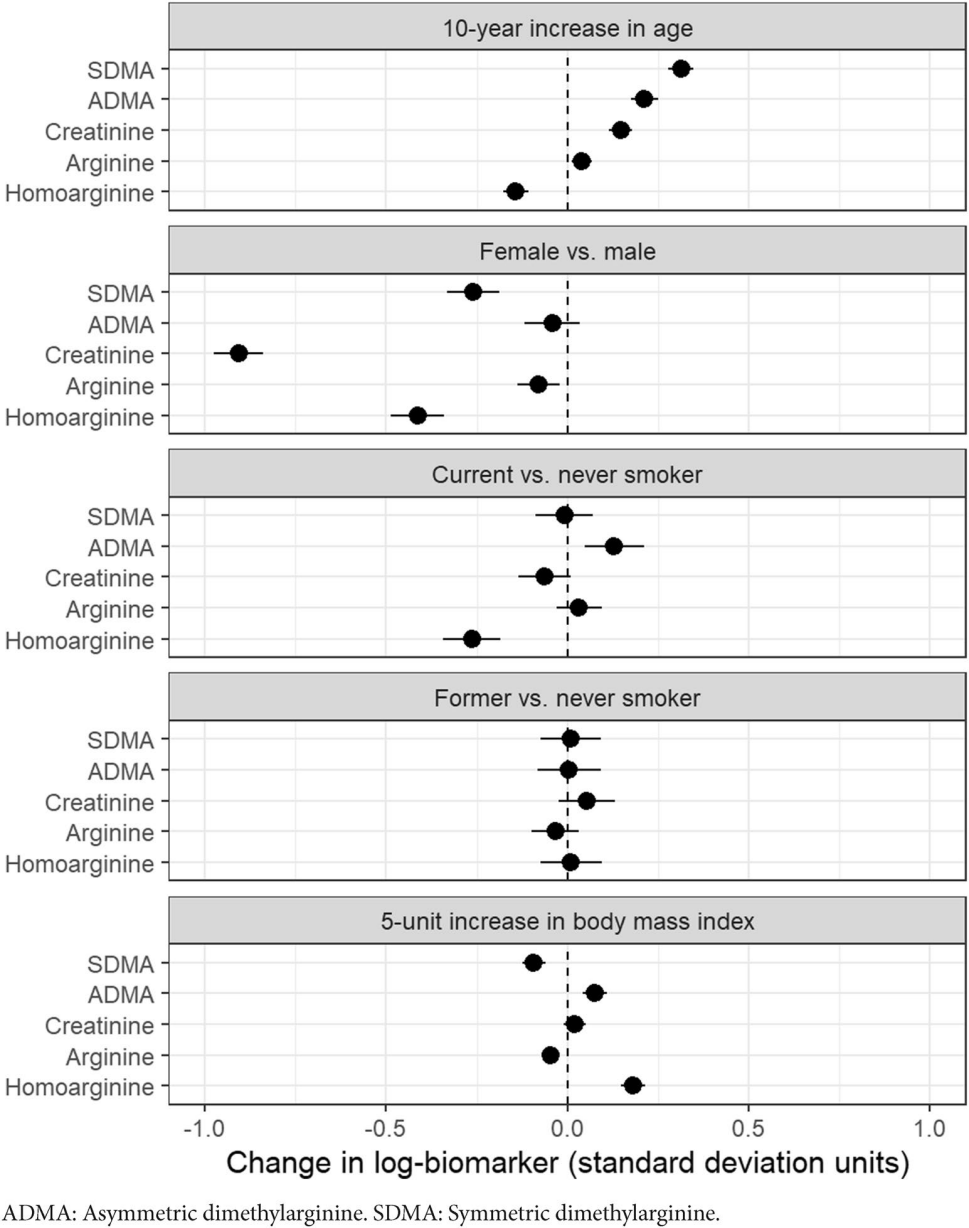
Differences in biomarkers of renal and endothelial function by age, sex, smoking status, and BMI in units of log-biomarker standard deviations, among 5167 control subjects in the Lung Cancer Cohort Consortium. Biomarkers are listed in order of the age coefficient.

Adjusted differences in standardized means of log-biomarker values are shown by age, sex, smoking status, and BMI for one-carbon metabolites in Fig. 1, for vitamins in Fig. 2, for kynurenine pathway metabolites and inflammation markers in Fig. 3, and for markers of renal and endothelial function in Fig. 4. Across all analyses, 93% (186/200) of analyses showed a difference lower than 0.25 standard-deviations, though most of these small differences were statistically significant due to large sample size. For 31 of 40 markers, the mean difference compared with never smokers was larger for current than for former smokers.

Age had a substantial impact on levels of most one-carbon metabolites (Fig. 1). Among all markers, the largest difference by age was for total cysteine (β = 0.40 standard-deviation increase per 10 years, 95%CI 0.36 to 0.43). Levels of one-carbon metabolites also varied strongly by sex, with markedly lower levels among women for betaine (β =  − 0.68, 95%CI − 0.74 to − 0.61), dimethylglycine (β =  − 0.51, 95%CI − 0.58 to − 0.44), total homocysteine (β =  − 0.38, 95%CI − 0.45 to − 0.31), and methionine (β =  − 0.38, 95%CI − 0.46 to − 0.30). Current smokers had higher levels of homocysteine than never smokers (β = 0.34, 95%CI 0.26 to 0.42). BMI did not have a strong influence on one carbon metabolite levels; the largest differences were β = 0.26 (for a 5-unit increase in BMI) for total cysteine (95%CI 0.23 to 0.29) and β =  − 0.25 for glycine (95% CI − 0.28 to − 0.22).

For vitamin biomarkers, most levels increased with age and were higher for women (Fig. 2). Current smokers had lower levels of almost all vitamins compared with never smokers, particularly B vitamins (β =  − 0.42, 95%CI − 0.50 to − 0.34 for vitamin B6/PLP, β =  − 0.35, 95%CI − 0.41 to − 0.28 for vitamin B9/folate, and β =  − 0.30, 95%CI − 0.38 to − 0.22 for vitamin B2/riboflavin). Differences between former and never smokers were much smaller, as were differences across BMI. One exception was vitamin E/gamma-tocopherol, which increased with BMI (β = 0.23 for a 5-unit increase in BMI, 95%CI 0.21 to 0.26).

With few exceptions, all kynurenine pathway and inflammation markers increased with age and BMI (Fig. 3). Among all the markers, the largest difference by BMI was for C-reactive protein (β = 0.38 standard-deviations per 5-kg/m^2^ increase, 95%CI 0.34 to 0.41). The largest differences were seen by sex, particularly for tryptophan (β =  − 0.47 for women, 95%CI − 0.55 to − 0.40). Current smokers had lower levels of almost every component of the kynurenine pathway; conversely, former smokers showed a small increase in all kynurenines compared with never smokers. CRP was higher in current smokers (β = 0.28, 95%CI 0.20 to 0.37) while differences between former and never smokers were small for all inflammation markers.

Of all markers and characteristics, the largest difference was for creatinine by sex (β =  − 0.91 for women, 95%CI − 0.98 to − 0.84; Fig. 4). Differences in creatinine by other variables were small. Markers of endothelial function showed inconsistent patterns by age and BMI, but were consistently decreased in women (e.g., β =  − 0.41 for homoarginine, 95%CI − 0.49 to − 0.34). Some differences in endothelial function markers were seen between current and never smokers, but not between former and never smokers.

We measured the prevalence of deficiency for 7 vitamins (Supplementary Table 5). Deficiency was most frequent for PLP (16%) and vitamin D (9%), but otherwise uncommon (0.2% to 3.4%). Stratified analysis of vitamin deficiencies showed more frequent PLP deficiency in men than in women (20% vs. 11%, *p* < 0.001), among individuals with lower BMI (decreasing from 39% among underweight individuals to 14% among obese individuals, *p* < 0.001), and with current smoking (25% vs. 7–8% for never and former smokers, *p* < 0.001). For vitamin D, deficiency decreased with age (decreasing from 11% among age 40–49 to 5% among age 70–80, *p* < 0.001). However, the mean change in blood Vitamin D levels with increasing age was small (β = 0.03, 95%CI − 0.01; 0.06, Fig. 2). Supplementary Tables 6 and 7 show that intake of multivitamins increased with age, and that vitamin D deficiency was lower among multivitamin users. Vitamin D deficiency also varied by sex, BMI, smoking status, and (for USA participants) race/ethnicity.

**Figure 5:**
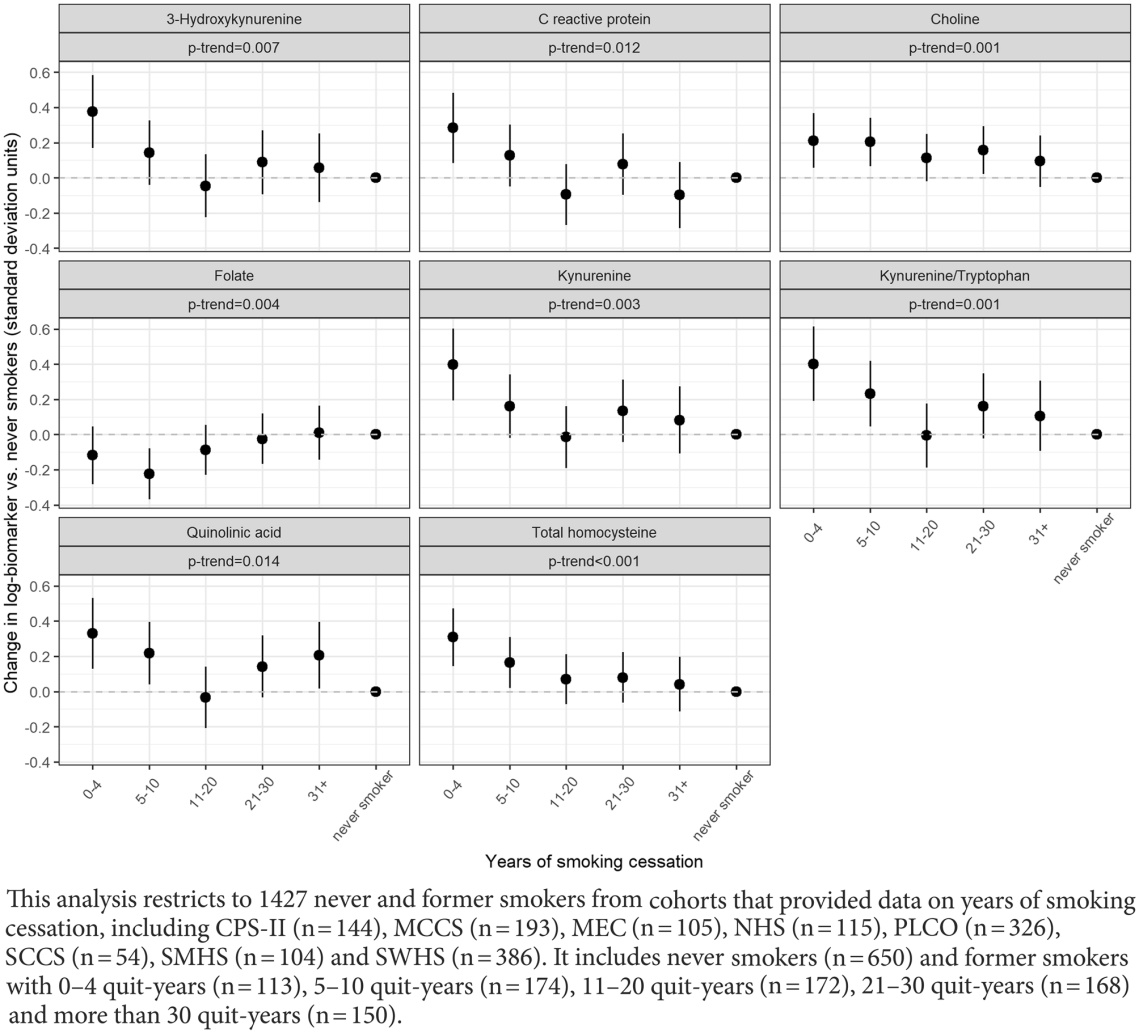
Trends in biomarker measurements among former smokers, by years of smoking cessation, for biomarkers with statistically significant trends (*p* < 0.05), among 1427 control subjects in selected cohorts from the Lung Cancer Cohort Consortium.

Trends in biomarker levels across time since smoking cessation were statistically significant (*p* < 0.05) for 8 of 40 biomarkers. Biomarkers with statistically significant trends are shown in Fig. 5, with the remainder shown in Supplementary Fig. 1. For some markers such as CRP, kynurenine, 3-hydroxykynurenine, and quinolinic acid, measurements appeared similar to those of never smokers within approximately 10–20 years of cessation (Fig. 5). For other markers, such as choline and total homocysteine, a steady trend was observed up to more than 30 years since quitting. This analysis was underpowered to detect biomarkers that normalize within 4 years of cessation, but visual inspection suggests that such markers might include kynurenic acid, pyridoxal, betaine, cystathionine, serine, and others (Supplementary Fig. 1).

Sensitivity analyses for model parameterization had negligible effects on coefficients (Supplementary Figs. 2, 3, and 4). The exclusion of outlying values of log-biomarkers had small effects (Supplementary Fig. 5). Similarly, the exclusion of single sex cohorts had very small effects on sex coefficients (Supplementary Fig. 6). Assuming a linear relationship between log-biomarker concentrations and age or BMI was generally justified as shown by the adjusted variable plots (Supplementary Figs. 7 and 8). The values and confidence intervals shown in Figs. 1, 2, 3, and 4 are provided in Supplementary Table 8.

## Discussion

In this study, we described variation in concentrations of 40 blood biomarkers across age, sex, smoking status, and BMI among cancer-free older adults. Most blood biomarkers of one-carbon metabolism, vitamin status, inflammation, and renal and endothelial function showed statistically robust but small variations across demographic and behavioral characteristics. There were important differences between men and women for several markers of one-carbon metabolism, some B vitamins, and components of the kynurenine pathway. Across multiple domains of physiological function, biomarker perturbations were stronger in current smokers than former smokers, illustrating the broadly positive influences of tobacco cessation.

Many of the biomarkers we studied were substantially altered in current smokers (compared with never smokers), whereas levels normalized with time since quitting smoking among former smokers. This observation suggests that changes in these biomarkers caused by smoking may resolve after smoking cessation. This pattern occurred broadly across multiple domains of analytes: for 31 out of 40 markers, the magnitude of difference between current and never smokers exceeded the difference between former and never smokers. These results are consistent with prior data for some individual markers, including vitamin E (alpha-tocopherol) and vitamin B12^18, 22^. Kynurenine pathway markers showed reductions among current smokers (vs. never smokers) but increases among former smokers, which is consistent with prior reports^23–26^. Decreased levels of kynurenines and neopterin in smokers might be attributable to decreased activity of cytokine IFN-gamma^27^.

Other studies have demonstrated that increased disease risk, particularly for lung cancer, can be reversed substantially with smoking cessation, and can even approach that of never smokers with sufficient quitting time^28, 29^. For example, levels of C-reactive protein revert to those of never-smokers after approximately 20 years of cessation^30^. Our data showed a similar result for CRP along with kynurenine, 3-hydroxykynurenine, and quinolinic acid. Some markers, such as choline and total homocysteine, may take even longer to normalize to the levels of never smokers, while others likely normalize within a few years. In general, our data suggest that this pattern of normalization after smoking cessation occurs across multiple physiological functions.

One-carbon metabolism is linked to B-vitamin status and is important for genome stability, with disturbances associated with tumorigenesis^31^. We found strong differences in some one-carbon metabolite levels between current and never smokers, which translated into higher prevalence of deficiency. We also observed lower levels of most one-carbon metabolites in women compared to men. This finding is consistent with some prior studies for total homocysteine^32^, betaine, and choline^33, 34^, though one study found the opposite pattern^35^.

Previous studies have addressed the role of several B-vitamins (B12, B9, B6), which are linked to one-carbon metabolism, in the development of lung cancer^9, 13, 36^. The fat-soluble vitamins A, D, and E are important for a variety of physiological functions relevant to potential associations between deficiencies and cancer ^7^. We found higher concentrations of most B-vitamins in women, although some studies showed the opposite pattern for PLP, folate^32^, cobalamin, and B6^34^. Previously reported sex differences in riboflavin levels^34, 37^ were not present in our data. One possible explanation is that women used more multivitamin supplements than men in our dataset (Supplementary Tables 6 and 7). For BMI, increasing vitamin E (gamma-tocopherol) levels with higher BMI, and decreasing vitamin D levels, are consistent with prior studies^38–40^. However, prevalence of vitamin D deficiency was highest among underweight individuals, followed by obese individuals. Vitamin D deficiency also decreased with age, while prior population-based studies in the USA showed potential small increases in vitamin D deficiency (or decreases in mean levels) with age^41, 42^.

Inflammation plays a central role in the pathogenesis of many chronic diseases, and many of the kynurenines are neuroactive compounds with immunomodulatory effects^23^. We observed expected increases in components of the kynurenine pathway and inflammation with increasing age^23^, and lower levels of kynurenine in women are also consistent with prior data. Positive associations of BMI with kynurenines and inflammation markers are consistent with prior findings for middle-aged individuals, with the exception of anthranilic acid^37, 43^.

Lower levels of creatinine in women are well-established, are due to lower amounts of muscle mass, and form the basis for sex-specific clinical reference ranges^44^. Unfortunately, we were not able to convert our creatinine measurements to estimated glomerular filtration rate (eGFR) because creatinine was not measured by isotope dilution mass spectrometry. Among this group of markers, we also observed lower levels of SDMA and homoarginine in women. Previous studies confirm the positive association between BMI and the endothelial function marker, ADMA^45^.

Strengths of our study include centralized biomarker analysis with robust quality control, participation of cohorts from different regions, and large sample size. The primary limitation is that we analyzed matched controls from a lung cancer study. This means that the study participants were not representative of their original cohorts, although this should not affect the relationships with biomarker concentrations estimated in our study, since we controlled for matching variables in analyses. In addition, our results may have limited generalizability to younger adults, as our population had a mean age of 62 years. Another limitation is that we had limited data on multivitamin use, precluding adjustment in models. Finally, we did not adjust *p*-values for multiple comparisons because the objective of our analyses was descriptive.

In summary, we analyzed population variation by age, sex, smoking status, and body mass index for multiple domains of biomarkers and metabolites including one-carbon metabolism, vitamin status, inflammation, and renal and endothelial function. Most markers showed small but statistically robust differences across these characteristics, although some larger differences were observed. This emphasizes the importance of adjusting for standard confounding factors in studies of pathways related to these biomarkers. Across multiple domains of physiological function, we observed resolution of biomarker perturbations in former compared with current smokers, illustrating the positive physiological effects of tobacco cessation.

## Supplementary Information

Supplementary Information 1.

## Data Availability

Data from the Lung Cancer Cohort Consortium are not publicly available because approval for analysis must be obtained from each participating cohort. Researchers who are interested in analyzing the LC3 data are encouraged to contact the corresponding author.
