# Supplementary material for: Epidemiology of 40 blood biomarkers of one-carbon metabolism, vitamin status, inflammation, and renal and endothelial function among cancer-free older adults
Source: Sci Rep. 2021 Jul 5;11:13805. doi: 10.1038/s41598-021-93214-8 (PMC8257595; doi:10.1038/s41598-021-93214-8)
Supplement: Supplementary file 1 — Supplementary Information 1. [file 41598_2021_93214_MOESM1_ESM.pdf]

SUPPLEMENT

Epidemiology of 40 blood biomarkers of one-carbon metabolism, vitamin status, inflammation, and renal and endothelial function among cancer-free older adults.

Hana Zahed[1], Mattias Johansson[1], Per M. Ueland[2], Øivind Midttun[3], Roger L. Milne[4,5,6], Graham G. Giles[4,5,6], Jonas Manjer[7,8], Malte Sandsveden[9], Arnulf Langhammer[10,11], Elin Pettersen Sørgjerd[12,13], Kjell Grankvist[14], Mikael Johansson[15], Neal D. Freedman[16], Wen-Yi Huang[16], Chu Chen[17], Ross Prentice[17], Victoria L. Stevens[18], Ying Wang[18], Loic Le Marchand[19], Lynne R. Wilkens[19], Stephanie J. Weinstein[16], Demetrius Albanes[16], Qiuyin Cai[20], William J. Blot[20], Alan A. Arslan[21,22,23], Anne Zeleniuch-Jacquotte[22,23], Xiao-Ou Shu[20], Wei Zheng[20], Jian-Min Yuan[24], Woon-Puay Koh[25], Kala Visvanathan[26], Howard D. Sesso[27,28], Xuehong Zhang[27,28], J Michael Gaziano[29,28], Anouar Fanidi[30], David Muller[31], Paul Brennan[1], Florence Guida[1], Hilary A. Robbins[1]

[1]Genomic Epidemiology Branch, International Agency For Research on Cancer, Lyon, France, [2]Department of Clinical Science, University of Bergen, Bergen, Norway, [3]Bevital AS, Bergen, Norway, [4]Cancer Epidemiology Division, Cancer Council Victoria, Melbourne, Australia, [5]Centre for Epidemiology and Biostatistics, School of Population and Global Health, The University of Melbourne, Melbourne, Australia, [6]Precision Medicine, School of Clinical Sciences at Monash Health, Monash University, Melbourne, Australia, [7]Department of Surgery, Skane University Hospital, Malmo, Sweden, [8]Lund University, Malmo, Sweden, [9]Department of Clinical Sciences Malmo, Lund University, Malmo, Sweden, [10]Department of Public Health and Nursing, Hunt Research Centre, Norwegian University of Science and Technology, Levanger, Norway, [11]Levanger Hospital , Nord-Trøndelag Hospital Trust, Levanger, Norway, [12]Department of Public Health and Nursing,NTNU, Hunt Research Centre, Norwegian University of Science and Technology, Levanger, Norway, [13]Department of Endocrinology, St. Olavs Hospital, Trondheim University Hospital, Levanger, Norway, [14]Department of Medical Biosciences, Umea University, Umea, Sweden, [15]Department of Radiation Sciences, Oncology, Umea University, Umea, Sweden, [16]Metabolic Epidemiology Branch, Division of Cancer Epidemiology and Genetics, National Cancer Institute, Bethesda, Maryland, USA, [17]Public Health Sciences Division, Fred Hutchinson Cancer Research Center, Seattle, USA, [18]American Cancer Society, Atlanta, USA, [19]University of Hawai'i Cancer Center, University of Hawaiʻi at Mānoa, Honolulu, USA, [20]Vanderbilt University Medical Center, Nashville, USA, [21]Department of Obstetrics and Gynecology, NYU Langone Health, New York, NY, USA, [22]Department of Population Health, NYU Langone Health, New York, NY, USA, [23]Perlmutter Comprehensive Cancer Center, NYU Langone Health, New York, NY, USA, [24]University of Pittsburgh Medical Center, Pittsburgh, USA, [25]Duke - NUS Medical School, Singapore, [26]Johns Hopkins Institute for Clinical and Translational Research, Baltimore, USA, [27]Brigham and Women's Hospital, Harvard Medical School, Boston, USA, [28]Harvard T.H. Chan School of Public Health, Boston, USA, [29]Brigham and Women's Hospital, Boston, USA, [30]Université Claude Bernard Lyon 1, Lyon, France, [31]Imperial College London School of Public Health, London, UK

Biomarker abbreviations used in Supplement:

AA = Anthranilic acid
ADMA = Asymmetric dimethylarginine
ARG = Arginine
ATOC = A-tocopherol
BET = Betaine
CHOL = Choline
COB = Cobalamin
CREAT = Creatinine
CRP = C reactive protein
CYSTA = Cystathionine
DMG = Dimethylglycine
FOL or SPFOLATE= Folate
GLY = Glycine
GTOC = G-tocopherol
HAA = 3-hydroxyanthranilic acid
HARG = Homoarginine,
HK = 3-Hydroxykynurenine
KA = Kynurenic acid
KYN = Kynurenine
KYN/TRP = Kynurenine tryptophan ratio
MET = Methionine
METSO = Methionine sulfoxide
MMA = Methylmalonic acid
NAM = Nicotinamide
NEOPT = Neopterin
PA = 4-Pyridoxic acid
Par = PAr index
PL = Pyridoxal
PLP = Pyridoxal 5'-phosphate
QA = Quinolinic acid
RIBO = Riboflavin
SARC = Sarcosine
SDMA = Symmetric dimethylarginine
SER = Serine
TCYS = Total cysteine
THCY = Total homocysteine
TRP = Tryptophan
Vit A or VA = Vitamin A
Vit D or VD = Vitamin D
XA = Xanthurenic acid

Supplementary methods: Statistical analyses for the primary analysis (main model) and sensitivity analyses.

Main model: linear mixed model with a fixed effect for cohort and random effect for batch

$$C_{biomarker} = \beta_0 + \beta_1 \times age + \beta_2 \times sex + \beta_3 \times BMI + \beta_4 \times I[formersmoker] + \beta_5 \times I[currentsmoker] + \beta_{6\_i} \times race_i + \beta_{7\_i} \times cohort_i + \beta_8 \times yearsofstorage + b_{Batch\_i} + \varepsilon$$

Sensitivity model 1: linear mixed model without cohort and a random effect for batch

$$C_{biomarker} = \beta_0 + \beta_1 \times age + \beta_2 \times sex + \beta_3 \times BMI + \beta_4 \times I[formersmoker] + \beta_5 \times I[currentsmoker] + \beta_{6\_i} \times race_i + \beta_7 \times yearsofstorage + b_{Batch\_i} + \varepsilon$$

Sensitivity model 2: linear mixed model with a fixed effect for cohort and without batch

$$C_{biomarker} = \beta_0 + \beta_1 \times age + \beta_2 \times sex + \beta_3 \times BMI + \beta_4 \times I[formersmoker] + \beta_5 \times I[currentsmoker] + \beta_{6\_i} \times race_i + \beta_{7\_i} \times cohort_i + \beta_8 \times yearsofstorage + \varepsilon$$

Sensitivity model 3: linear mixed model with a fixed effect for cohort and random effect for batch, without the adjustment for storage time

$$C_{biomarker} = \beta_0 + \beta_1 \times age + \beta_2 \times sex + \beta_3 \times BMI + \beta_4 \times I[formersmoker] + \beta_5 \times I[currentsmoker] + \beta_{6\_i} \times race_i + \beta_{7\_i} \times cohort_i + b_{Batch\_i} + \varepsilon$$

Supplementary Table 1: Laboratory methods used for biomarker measurements

Method used	Biomarkers measured
Liquid chromatography (LC)-MS/MS	RIBO, PLP, PL, PA, KA, AA, HK, XA, HAA, NEOPT, CHOL, BET, DMG, CREAT, METSO, ARG, ADMA, SDMA, VIT A (all trans retinol), VIT D, ATOC, GTOC, QA, NAM, HARG
Gas chromatography (GC)-MS/MS	MMA, THCY, TCYS, MET, SER, GLY, CYSTA, SARC, TRP, KYN
MALDI-TOF MS	CRP
Microbiologic methods	COB, FOL

This information can be found on the official website of BEVITAL AS Laboratory at: https://folk.uib.no/mfapu/Pages/BV/BVSite/platf.html

Supplementary Table 2: Years of blood collection in each participating cohort

Cohort	Years of blood collection	Number of years of blood collection	Median storage time
NSHDS	1989 to 2010	21	13
HUNT	1995 to 2008	13	14
PLCO	1994 to 2006	12	9
SCHS	1994 to 2005	11	8
MEC	1996 to 2006	10	7
SCCS	2002 to 2008	6	6
MDCS	1991 to 1996	5	16
NYU	1985 to 1990	5	24
PHS	1995 to 2000	5	14
WHI*	1993 to 1998	5	15
MCCS	1990 to 1994	4	17.5
SMHS	2002 to 2006	4	6
ATBC	1985 to 1988	3	23
CPS	1998 to 2001	3	10
SCS	1986 to 1989	3	22
SWHS	1997 to 2000	3	12
HPFS	1993 to 1995	2	17
WHS	1993 to 1995	2	17
NHS	1989 to 1990	1	20
CLUE	1989 to 1989	0	21

*Individual years of blood draw were not provided for WHI. We imputed 1995 for the year of blood draw for all WHI participants in the regression models. Storage time for WHI was imputed to 15 years.

Supplementary Table 3: Descriptive characteristics of control participants in the Lung Cancer Cohort Consortium, stratified by cohort

		Gender		Race				Smoking Status		
**Cohort**	**N**	**Female**	**Male**	**White**	**Black**	**Asian**	**Other**	**Current**	**Former**	**Never**
**ATBC**	200	0 (0%)	200 (100%)	200 (100%)	0 (0%)	0 (0%)	0 (0%)	200 (100%)	0 (0%)	0 (0%)
**CLUE**	171	70 (40.9%)	101 (59.1%)	170 (99.4%)	1 (0.6%)	0 (0%)	0 (0%)	85 (49.7%)	69 (40.4%)	17 (9.9%)
**CPS**	179	98 (54.7%)	81 (45.3%)	176 (98.3%)	1 (0.6%)	2 (1.1%)	0 (0%)	35 (19.6%)	105 (58.7%)	39 (21.8%)
**HPFS**	130	0 (0%)	130 (100%)	130 (100%)	0 (0%)	0 (0%)	0 (0%)	37 (28.5%)	77 (59.2%)	16 (12.3%)
**HUNT**	174	118 (67.8%)	56 (32.2%)	174 (100%)	0 (0%)	0 (0%)	0 (0%)	113 (64.9%)	40 (23%)	21 (12.1%)
**MCCS**	358	140 (39.1%)	218 (60.9%)	358 (100%)	0 (0%)	0 (0%)	0 (0%)	163 (45.5%)	149 (41.6%)	46 (12.8%)
**MDCS**	199	109 (54.8%)	90 (45.2%)	199 (100%)	0 (0%)	0 (0%)	0 (0%)	78 (39.2%)	71 (35.7%)	50 (25.1%)
**MEC**	148	57 (38.5%)	91 (61.5%)	99 (66.9%)	0 (0%)	49 (33.1%)	0 (0%)	43 (29.1%)	74 (50%)	31 (20.9%)
**NHS**	328	328 (100%)	0 (0%)	284 (86.6%)	0 (0%)	0 (0%)	44 (13.4%)	166 (50.6%)	122 (37.2%)	40 (12.2%)
**NSHDS**	230	115 (50%)	115 (50%)	129 (56.1%)	0 (0%)	0 (0%)	101 (43.9%)	128 (55.7%)	74 (32.2%)	28 (12.2%)
**NYU**	167	167 (100%)	0 (0%)	145 (86.8%)	10 (6%)	0 (0%)	12 (7.2%)	69 (41.3%)	69 (41.3%)	29 (17.4%)
**PHS**	76	0 (0%)	76 (100%)	76 (100%)	0 (0%)	0 (0%)	0 (0%)	8 (10.5%)	43 (56.6%)	25 (32.9%)
**PLCO**	440	134 (30.5%)	306 (69.5%)	414 (94.1%)	26 (5.9%)	0 (0%)	0 (0%)	107 (24.3%)	284 (64.5%)	49 (11.1%)
**SCCS**	209	68 (32.5%)	141 (67.5%)	0 (0%)	209 (100%)	0 (0%)	0 (0%)	155 (74.2%)	40 (19.1%)	14 (6.7%)
**SCHS**	409	125 (30.6%)	284 (69.4%)	0 (0%)	0 (0%)	409 (100%)	0 (0%)	210 (51.3%)	83 (20.3%)	116 (28.4%)
**SCS**	502	0 (0%)	502 (100%)	0 (0%)	0 (0%)	502 (100%)	0 (0%)	440 (87.6%)	11 (2.2%)	51 (10.2%)
**SMHS**	419	0 (0%)	419 (100%)	0 (0%)	0 (0%)	419 (100%)	0 (0%)	300 (71.6%)	70 (16.7%)	49 (11.7%)
**SWHS**	417	417 (100%)	0 (0%)	0 (0%)	0 (0%)	417 (100%)	0 (0%)	30 (7.2%)	5 (1.2%)	382 (91.6%)
**WHI**	228	228 (100%)	0 (0%)	215 (94.3%)	0 (0%)	13 (5.7%)	0 (0%)	0 (0%)	0 (0%)	228 (100%)
**WHS**	183	183 (100%)	0 (0%)	178 (97.3%)	0 (0%)	1 (0.5%)	4 (2.2%)	83 (45.4%)	67 (36.6%)	33 (18%)

Supplementary Table 4: Effects of storage time on log biomarker concentrations, per 1 year of storage.

Change in log-biomarker (standard deviation unit)	Beta (95%CI or IQR)	Biomarker
Minimum	-0.001 (-0.015; 0.012)	Neopterin
Maximum	0.038 (0.024; 0.051)	Vitamin E- gamma tocopherol
Median	0.014 (0.006; 0.021)	NA

Effects of storage time on biomarker concentrations were extracted after fitting a linear regression model for each biomarker against storage time, adjusted for cohort. To determine the minimum, maximum, mean, and median, the absolute value of the coefficient estimates was considered, but the coefficient is listed as-is (without taking the absolute value). Biomarker samples were stored and frozen for up to 25 years after blood draw. Median storage time was 14 years for all biomarkers among all cohorts with an IQR of 9 years to 20 years.

Supplementary Figure 1: Trends in biomarker measurements by years of smoking cessation, for biomarkers without statistically significant trends

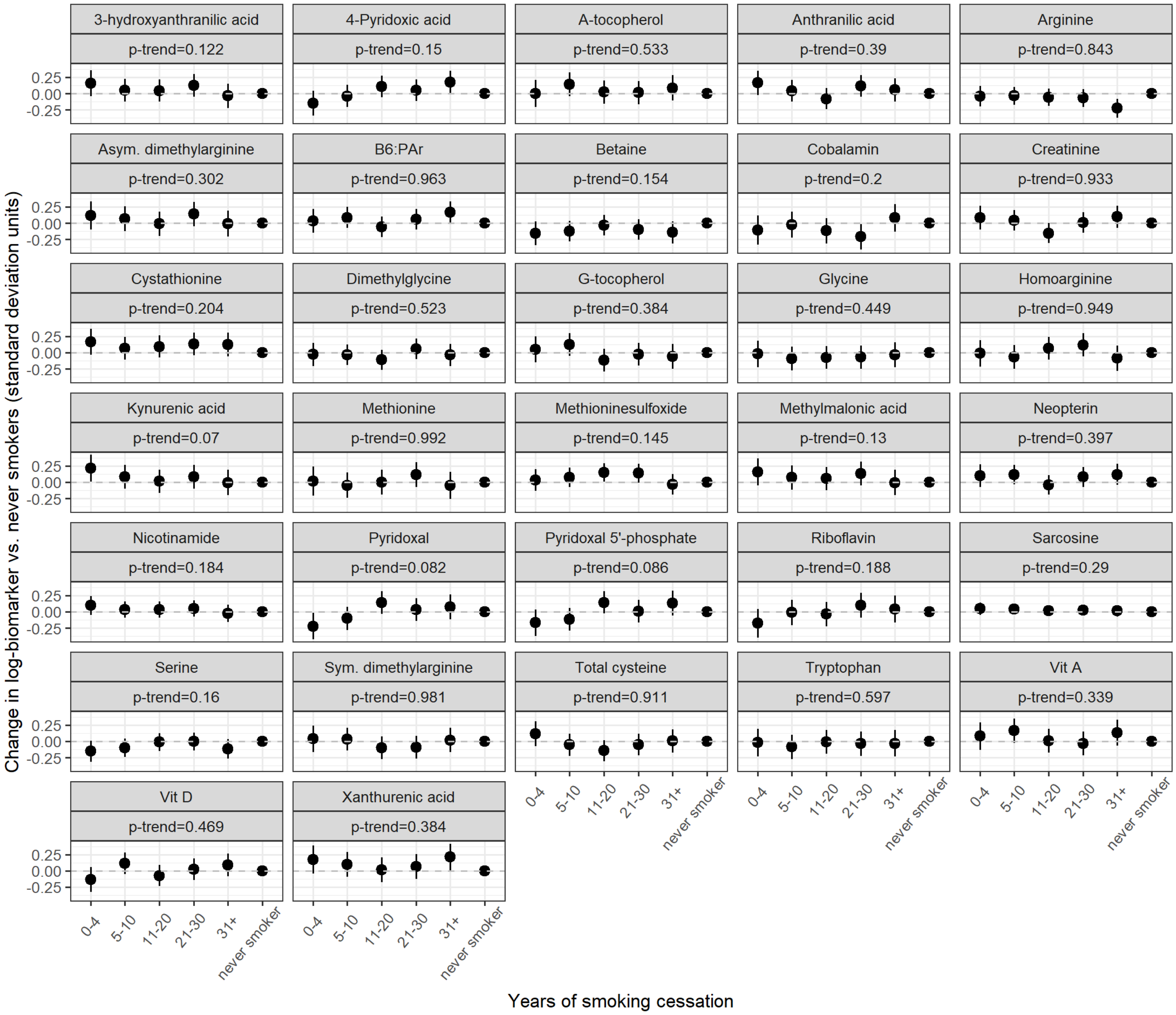

This analysis restricts to 1,427 never and former smokers from cohorts that provided data on years of smoking cessation, including CPS-II (n=144), MCCS (n=193), MEC (n=105), NHS (n=115), PLCO (n=326), SCCS (n=54), SMHS (n=104) and SWHS (n=386). It includes never smokers (n=650) and former smokers with 0-4 quit-years (n=113), 5-10 quit-years (n=174), 11-20 quit-years (n=172), 21-30 quit-years (n=168) and more than 30 quit-years (n=150).

Supplementary Table 5: Description of vitamin deficiencies among 5,167 control subjects in the Lung Cancer Cohort Consortium

		Prevalence of deficiency, N (%)						
Characteristic	N	Riboflavin 5 nmol/L*	PLP 20 nmol/L*	Folate 5 nmol/L*	Cobalamin 150 pmol/L*	Vitamin A 0.7 μmol/L*	Vitamin E (α-tocopherol) 12 μmol/L*	Vitamin D 30 nmol/L*
Total	5167	174 (3.4)	826 (16.0)	98 (1.9)	41 (0.8)	9 (0.2)	14 (0.3)	462 (9.0)
Age								
40-49	473	28 (5.9)	62 (13.1)	16 (3.4)	2 (0.4)	2 (0.4)	3 (0.6)	53 (11.2)
50-59	1448	61 (4.2)	302 (20.9)	41 (2.8)	9 (0.6)	2 (0.1)	4 (0.3)	155 (10.7)
60-69	2324	62 (2.7)	362 (15.6)	27 (1.2)	19 (0.8)	3 (0.1)	3 (0.1)	209 (9.0)
70-80	922	23 (2.5)	100 (10.8)	14 (1.5)	11(1.2)	2 (0.2)	4 (0.4)	45 (4.9)
p-het		**0.001**	**<0.001**	**<0.001**	0.39	0.39	0.11	**<0.001**
Gender								
Male	2810	104 (3.7)	562 (20.0)	49 (1.7)	24 (0.9)	5 (0.2)	8 (0.3)	265 (9.4)
Female	2357	70 (3)	264 (11.2)	49 (2.1)	17 (0.7)	4 (0.2)	6 (0.3)	197 (8.4)
p-het		0.16	**<0.001**	0.41	0.64	0.99	0.99	**<0.001**
BMI								
Underweight	121	3 (2.5)	47 (38.8)	2 (1.7)	1 (0.8)	1 (0.8)	1 (0.8)	22 (18.2)
Normal	2608	85 (3.3)	477 (18.3)	52 (2.0)	17 (0.7)	4 (0.2)	11 (0.4)	241 (9.2)
Overweight	1802	68 (3.8)	214 (11.9)	34 (1.9)	17 (0.9)	3 (0.2)	2 (0.1)	128 (7.1)
Obese	636	18 (2.8)	88 (13.8)	10 (1.6)	6 (0.9)	1 (0.2)	0 (0.0)	71 (11.2)
p-het		0.67	**<0.001**	0.95	0.58	0.36	**0.05**	**<0.001**
Smoking status								
Never	1264	44 (3.5)	92 (7.3)	12 (0.9)	11 (0.9)	2 (0.2)	3 (0.2)	117 (9.3)
Former	1453	24 (1.7)	122 (8.4)	15 (1.0)	13 (0.9)	2 (0.1)	2 (0.1)	66 (4.5)
Current	2450	106 (4.3)	612 (25.0)	71 (2.9)	17 (0.7)	5 (0.2)	9 (0.4)	279 (11.4)
p-het		**<0.001**	**<0.001**	**<0.001**	0.71	0.99	0.44	**<0.001**
Race (USA)								
White	1887	15 (0.8)	170 (9.0)	8 (0.4)	14 (0.7)	1 (0.1)	0 (0.0)	77 (4.1)
Black	247	16 (6.5)	27 (10.9)	1 (0.4)	3 (1.2)	3 (1.2)	3 (1.2)	78 (31.6)
p-het		**<0.001**	**0.005**	0.07	0.41	**0.03**	**0.006**	**<0.001**

*Cutpoint for defining deficiency, as described in Midttun et al, Am J Clin Nutr 2017.
We calculated p-values for heterogeneity (p-het) using the Fisher exact test. P-values less than 0.05 are shown in bold. We did not adjust for multiple comparisons. PLP: pyridoxal 5'-phosphate.

Supplementary Figure 2: Model parameterization sensitivity analyses. Effect of removing cohort adjustment on coefficients measuring the association between 40 biomarkers and age, sex, smoking status, and BMI

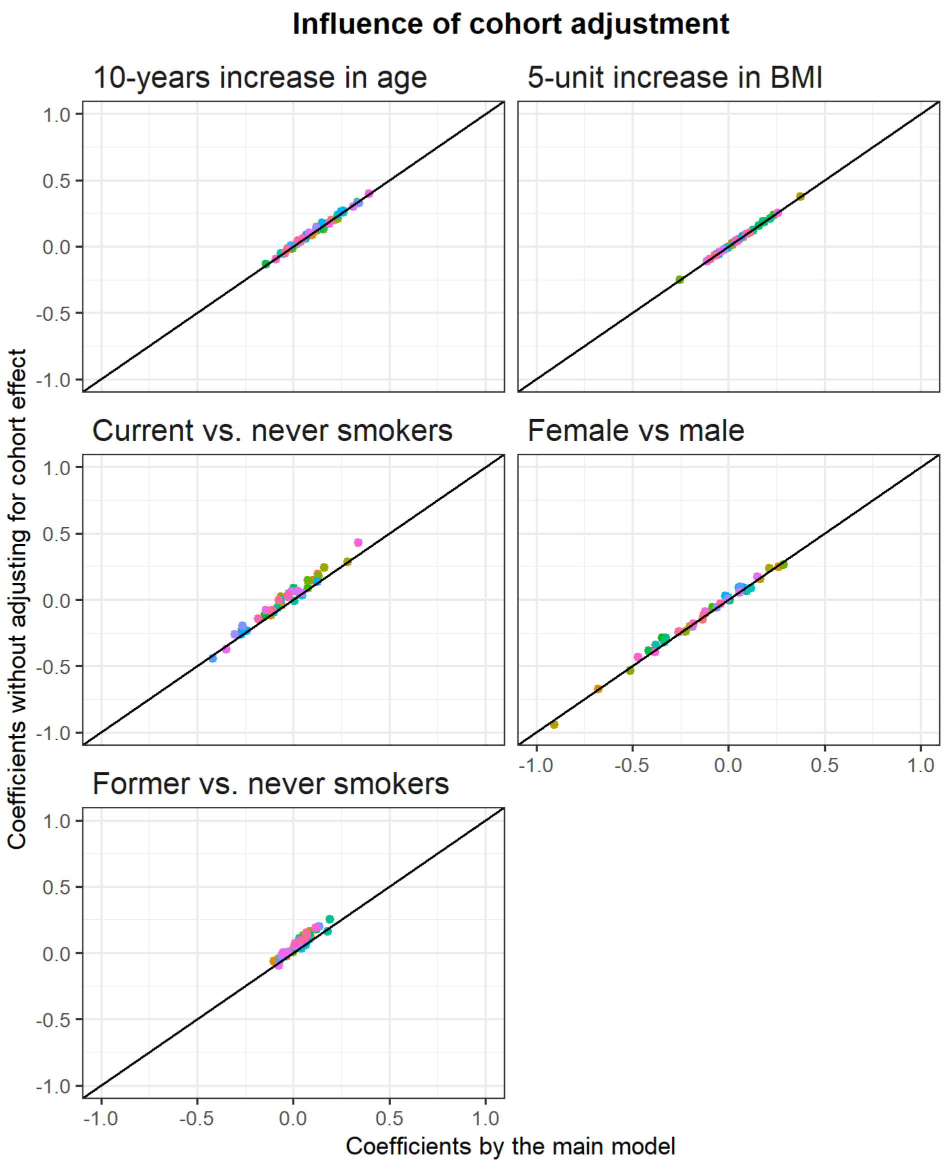

Each point represents the coefficients generated for one biomarker.

Supplementary Figure 3: Model parameterization sensitivity analyses. Effect of removing random effects for batch adjustment on coefficients measuring the association between 40 biomarkers and age, sex, smoking status, and BMI

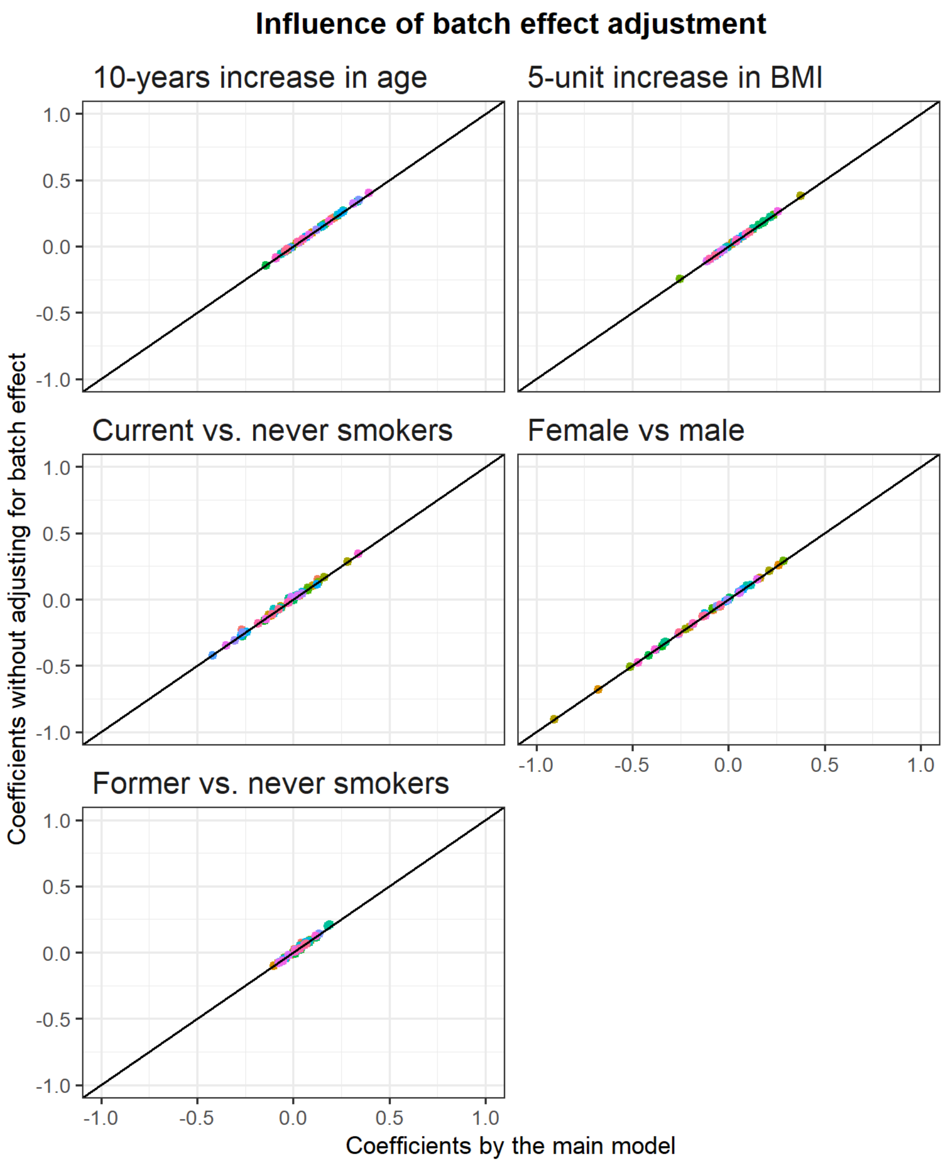

Each point represents the coefficients generated for one biomarker.

Supplementary Figure 4: Model parameterization sensitivity analyses. Effect of removing time storage adjustment on coefficients measuring the association between 40 biomarkers and age, sex, smoking status and BMI

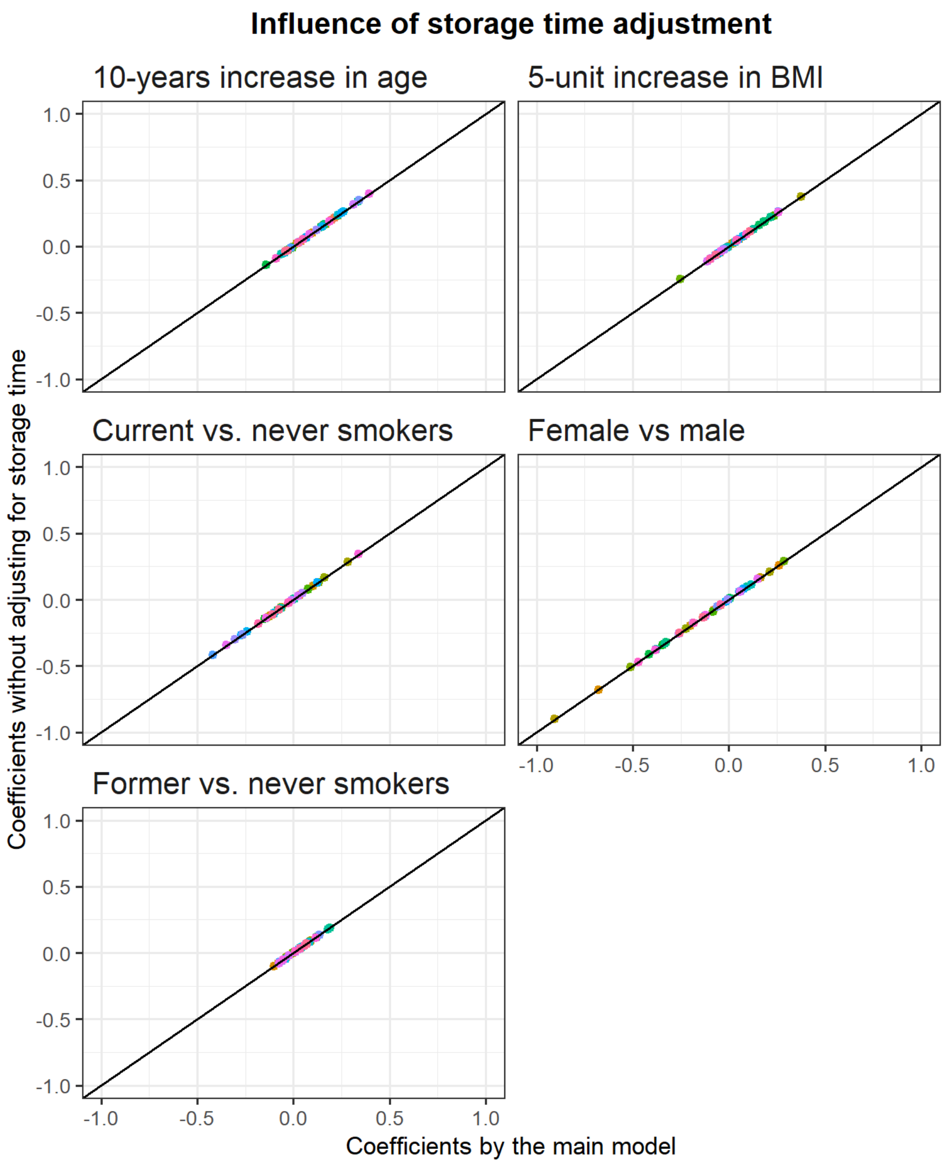

Each point represents the coefficients generated for one biomarker.

Supplementary Figure 5: Model parameterization sensitivity analyses. Effect of removing outliers on coefficients measuring the association between 40 biomarkers and age, sex, smoking status, and BMI

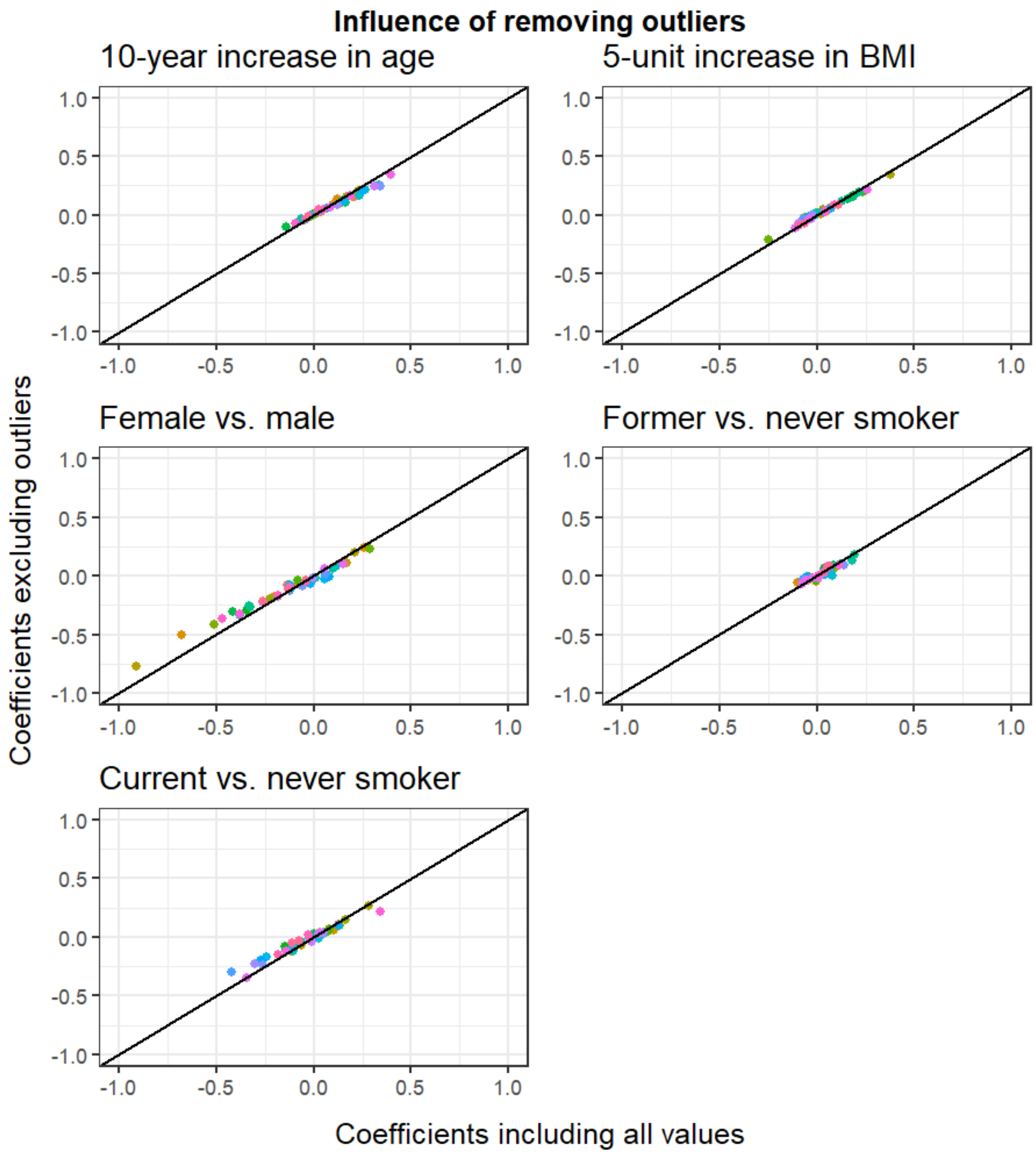

Each point represents the coefficients generated for one biomarker.
We defined outliers as values with an absolute difference with the mean concentration of a biomarker greater than 1.5 times the interquartile range.

Supplementary Figure 6: Effect of excluding single-sex cohorts on the coefficients measuring associations between 40 biomarkers and gender.

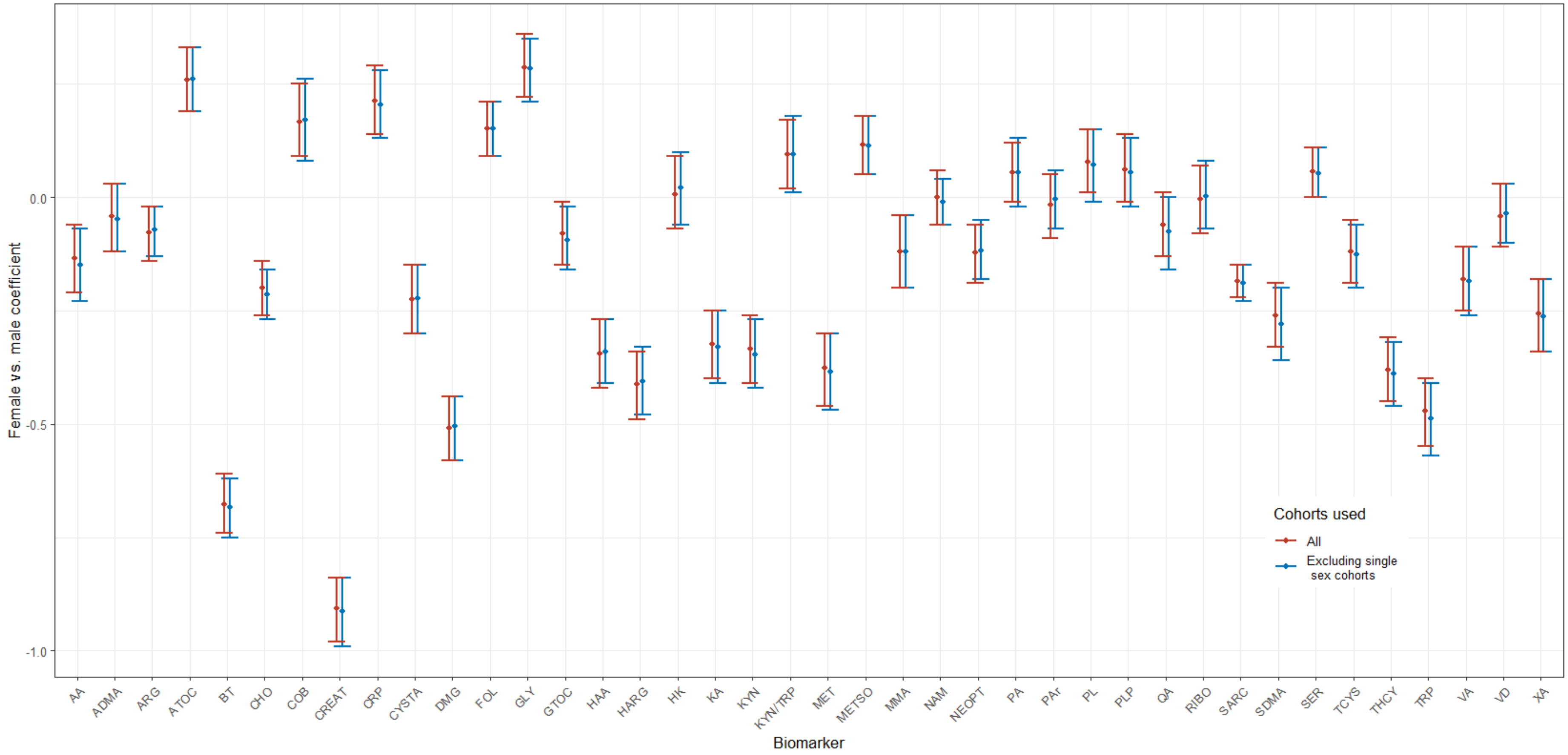

Single-sex cohorts include male-only cohorts (ATBC, HPFS, PHS, SCS, SMHS) and female-only cohorts (NHS, NYU, SWHS, WHI, WHS).

Supplementary Figure 7: Adjusted variable plots to examine the assumption of a linear relationship between each biomarker and age (see list of abbreviations).

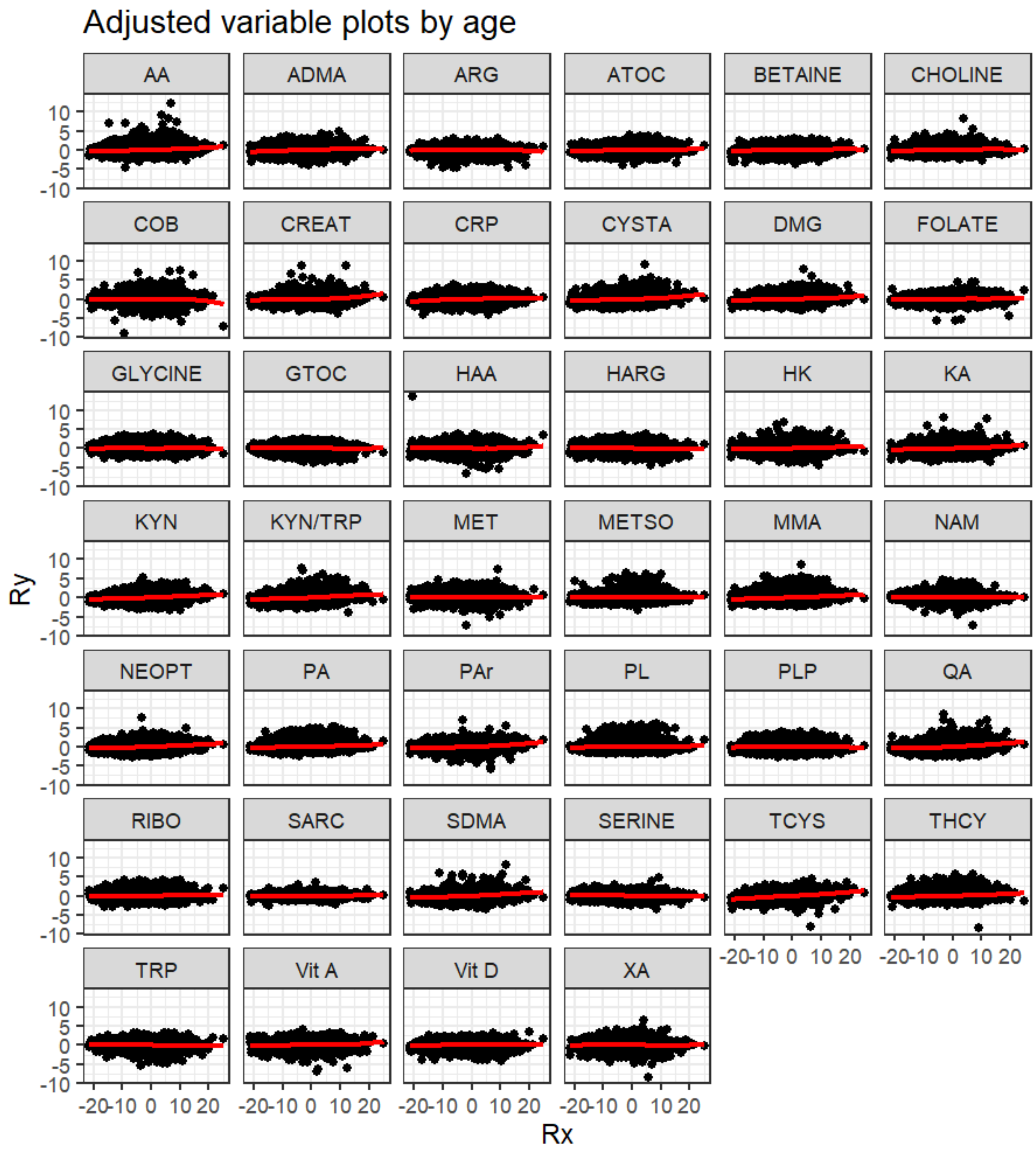

We used a general linear model to regress biomarker's concentration on every predictor used in the main model except for age (i.e. sex, BMI, smoking status, race, cohort, and storage time). The residuals from this regression (Rx) contain information about biomarker concentration that is not explained by those predictors. We separately used a general linear model to regress age against predictors used in the previous model. The residuals from this regression (Ry) contain information about age not explained by the other predictors. Finally, we plotted Rx against Ry to visualize the relationship between the biomarker and age after adjusting for all other predictors. The relationship is shown by a smoothed LOESS curve.

Supplementary Figure 8: Adjusted variable plots to examine the assumption of a linear relationship between each biomarker and BMI (see list of abbreviations).

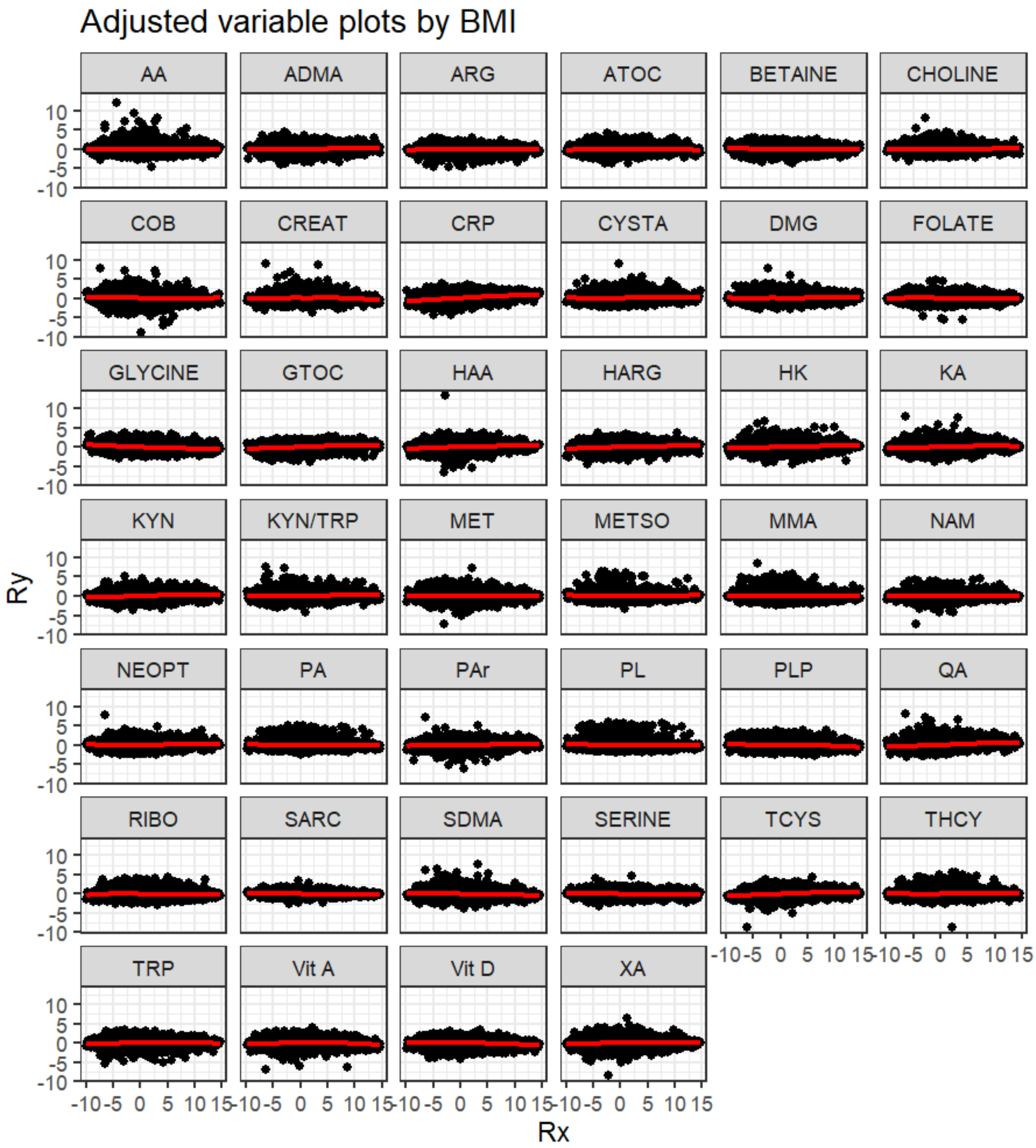

We used a general linear model to regress biomarker's concentration on every predictor used in the main model except for BMI (i.e. sex, age, smoking status, race, cohort, and storage time). The residuals from this regression (Rx) contain information about biomarker concentration that is not explained by those predictors. We separately used a general linear model to regress BMI against predictors used in the previous model. The residuals from this regression (Ry) contain information about BMI not explained by the other predictors. Finally, we plotted Rx against Ry to visualize the relationship between the biomarker and BMI after adjusting for all other predictors. The relationship is shown by a smoothed LOESS curve.

Supplementary Table 6: Prevalence of multivitamin use across demographic variables.

	Multivitamin use if measured as current or no current use, N current user / total respondents (%)	Multivitamin use if measured as ever or never use, N ever user / total respondents (%)
**Age, years**		
<50	64/310 (20.6)	27/38 (71.1)
50-60	139/691 (20.1)	73/142 (51.4)
60-70	278/1184 (23.5)	119/374 (31.8)
70-80	147/425 (34.6)	89/259 (34.4)
p-value	<0.001	<0.001
**Gender**		
Male	215/1108 (19.4)	111/421 (26.4)
Female	413/1502 (27.5)	197/392 (50.3)
p-value	<0.001	<0.001
**BMI**		
Underweight	8/37 (21.6)	3/5 (60.0)
Normal	299/1225 (24.4)	158/355 (44.5)
Overweight	224/976 (23.0)	106/316 (33.5)
Obese	97/372 (26.1)	41/137 (29.9)
p-value	0.64	0.003
**Smoking status**		
Never	183/856 (21.4)	61/135 (45.2)
Former	222/698 (31.8)	139/442 (31.4)
Current	223/1056 (21.1)	108/236 (45.8)
p-value	<0.001	<0.001
**Race**		
White	485/1492 (32.5)	266/722 (36.8)
Black	61/210 (29.0)	7/28 (25.0)
Asian	61/851 (7.2)	28/50 (56.0)
Other	21/57 (36.8)	7/13 (53.8)
p-value	<0.001	0.01

Multivitamin supplement use was reported as follows: never or ever use in MEC, NYUWHS, PHS, and PLCO; current or no current use in CLUE, CPS-II, HPFS, HUNT, MCCS, NHS, NSHDS, SCCS, SMHS, SWHS, and WHI; and a mixture of these two systems in WHS. No information on multivitamin supplement use was reported in ATBC, MDCS, SCHS and SCS. Overall, information on multivitamin use was missing in 1744 of 5164 participants.

Supplementary Table 7: Prevalence of vitamin deficiencies by multivitamin use.

Multivitamin use	Vitamin D deficiency N (%)	PLP (B6) deficiency N (%)	Folate (B9) deficiency N (%)
Never users	22/505 (4.4)	67/505 (13.3)	1/505 (0.2)
Ever users	6/308 (2.0)	8/308 (2.6)	0/308 (0.0)
p-value	0.10	<0.001	--
No current users	268/1977 (13.6)	228/1982 (11.5)	36/1982 (1.8)
Current users	29/628 (4.6)	24/628 (3.8)	8/628 (1.3)
p-value	<0.001	<0.001	0.46

Some cohorts measured multivitamin use as ever/never, while others measured as current/no current use. Cutpoints used to indicate vitamin deficiency were 20 nmol/L for PLP, 5 nmol/L for folate, and 30 nmol/L for vitamin D (Midttun et al, Am J Clin Nutr 2017).

Supplementary Table 8: Adjusted differences in standardized means of log-biomarker values. These values are shown in the main manuscript in Figures 1, 2, 3, and 4.

Biomarkers related to	10-year increase in age	Female vs. male	Former vs. never smoker	Current vs. never smoker	5-unit increase in body mass index
**One carbon metabolism**					
Total cysteine	0.40 (0.36; 0.43)	-0.12 (-0.19; -0.05)	-0.05 (-0.13; 0.03)	-0.14 (-0.22; -0.07)	0.26 (0.23; 0.29)
Cystathionine	0.23 (0.20; 0.27)	-0.22 (-0.30; -0.15)	0.09 (0.00; 0.17)	0.16 (0.08; 0.24)	0.13 (0.10; 0.16)
Total homocysteine	0.19 (0.15; 0.22)	-0.38 (-0.45; -0.31)	0.12 (0.04; 0.20)	0.34 (0.26; 0.42)	0.05 (0.01; 0.08)
Dimethylglycine	0.16 (0.13; 0.19)	-0.51 (-0.58; -0.44)	-0.04 (-0.11; 0.04)	0.13 (0.06; 0.20)	0.03 (0.00; 0.06)
Choline	0.12 (0.09; 0.15)	-0.20 (-0.26; -0.14)	0.09 (0.02; 0.16)	0.10 (0.04; 0.17)	0.05 (0.03; 0.08)
Betaine	0.10 (0.07; 0.13)	-0.68 (-0.74; -0.61)	-0.10 (-0.17; -0.03)	-0.06 (-0.13; 0.01)	-0.06 (-0.09; -0.03)
Methionine sulfoxide	0.07 (0.04; 0.10)	0.12 (0.05; 0.18)	0.04 (-0.04; 0.11)	-0.10 (-0.17; -0.03)	-0.01 (-0.04; 0.02)
Sarcosine	0.03 (0.01; 0.04)	-0.18 (-0.22; -0.15)	0.03 (-0.01; 0.08)	0.05 (0.01; 0.09)	-0.03 (-0.04; -0.01)
Glycine	-0.01 (-0.04; 0.03)	0.29 (0.22; 0.36)	-0.05 (-0.13; 0.03)	0.08 (0.00; 0.15)	-0.25 (-0.28; -0.22)
Methionine	-0.06 (-0.10; -0.02)	-0.38 (-0.46; -0.30)	0.03 (-0.06; 0.12)	-0.06 (-0.15; 0.02)	0.00 (-0.04; 0.03)
Serine	-0.09 (-0.12; -0.06)	0.06 (0.00; 0.11)	-0.02 (-0.09; 0.04)	0.02 (-0.04; 0.09)	-0.11 (-0.14; -0.09)
**Vitamin status**					
B12: Methylmalonic acid	0.23 (0.19; 0.27)	-0.12 (-0.20; -0.04)	0.08 (-0.01; 0.17)	0.02 (-0.06; 0.11)	-0.05 (-0.09; -0.02)
B6:4-Pyridoxic acid	0.15 (0.12; 0.18)	0.05 (-0.01; 0.12)	-0.04 (-0.12; 0.04)	-0.24 (-0.32; -0.17)	-0.03 (-0.06; 0.01)
B2: Riboflavin	0.12 (0.09; 0.16)	0.00 (-0.08; 0.07)	-0.06 (-0.15; 0.03)	-0.30 (-0.38; -0.22)	-0.02 (-0.06; 0.01)
VE: A-tocopherol	0.12 (0.09; 0.15)	0.26 (0.19; 0.33)	-0.01 (-0.08; 0.07)	-0.12 (-0.19; -0.04)	0.02 (-0.01; 0.05)
B9: Folate	0.08 (0.05; 0.12)	0.15 (0.09; 0.21)	-0.08 (-0.15; 0.00)	-0.35 (-0.41; -0.28)	-0.04 (-0.07; -0.01)
B6: Pyridoxal	0.07 (0.03; 0.10)	0.08 (0.01; 0.15)	-0.05 (-0.13; 0.03)	-0.27 (-0.35; -0.19)	-0.06 (-0.09; -0.03)
Vitamin A	0.05 (0.01; 0.09)	-0.18 (-0.25; -0.11)	0.06 (-0.03; 0.14)	-0.03 (-0.10; 0.05)	0.04 (0.01; 0.07)
Vitamin D	0.03 (-0.01; 0.06)	-0.04 (-0.11; 0.03)	0.04 (-0.05; 0.12)	-0.18 (-0.26; -0.10)	-0.10 (-0.13; -0.06)
B12: Cobalamin	0.02 (-0.02; 0.06)	0.17 (0.09; 0.25)	-0.08 (-0.17; 0.01)	-0.13 (-0.21; -0.04)	-0.06 (-0.10; -0.03)
B6: Pyridoxal 5'-phosphate	-0.01 (-0.05; 0.02)	0.06 (-0.01; 0.14)	-0.07 (-0.15; 0.01)	-0.42 (-0.50; -0.34)	-0.07 (-0.10; -0.04)
VE: G-tocopherol	-0.01 (-0.04; 0.03)	-0.08 (-0.15; -0.01)	0.00 (-0.08; 0.07)	0.08 (0.00; 0.15)	0.23 (0.21; 0.26)
B3: Nicotinamide	-0.05 (-0.07; -0.02)	0.00 (-0.06; 0.06)	0.04 (-0.02; 0.11)	0.05 (-0.01; 0.11)	0.03 (0.01; 0.06)
**Kynurenine pathway and inflammation**					
Quinolinic acid	0.34 (0.31; 0.38)	-0.06 (-0.13; 0.01)	0.14 (0.05; 0.22)	-0.26 (-0.34; -0.18)	0.26 (0.23; 0.29)

Kynurenine/Tryptophan	0.33 (0.30; 0.37)	0.09 (0.02; 0.17)	0.18 (0.09; 0.27)	0.01 (-0.08; 0.09)	0.13 (0.10; 0.16)
Kynurenine	0.26 (0.23; 0.30)	-0.33 (-0.41; -0.26)	0.19 (0.11; 0.27)	-0.02 (-0.10; 0.06)	0.22 (0.18; 0.25)
Neopterin	0.26 (0.23; 0.29)	-0.12 (-0.19; -0.06)	0.07 (-0.01; 0.14)	-0.10 (-0.17; -0.03)	0.07 (0.05; 0.10)
B6:PAr	0.25 (0.22; 0.28)	-0.02 (-0.09; 0.05)	0.03 (-0.05; 0.11)	0.13 (0.05; 0.20)	0.05 (0.02; 0.08)
Anthranilic acid	0.20 (0.16; 0.24)	-0.13 (-0.21; -0.06)	0.04 (-0.05; 0.13)	-0.27 (-0.35; -0.18)	0.00 (-0.04; 0.03)
C reactive protein	0.17 (0.13; 0.20)	0.21 (0.14; 0.29)	0.03 (-0.05; 0.12)	0.28 (0.20; 0.37)	0.38 (0.34; 0.41)
Kynurenic acid	0.16 (0.12; 0.20)	-0.32 (-0.40; -0.25)	0.08 (0.00; 0.17)	-0.08 (-0.17; 0.00)	0.19 (0.15; 0.22)
3-Hydroxykynurenine	0.14 (0.10; 0.18)	0.01 (-0.07; 0.09)	0.12 (0.03; 0.21)	0.00 (-0.08; 0.09)	0.16 (0.12; 0.19)
3-hydroxyanthranilic acid	-0.03 (-0.06; 0.01)	-0.34 (-0.42; -0.27)	0.04 (-0.05; 0.13)	-0.15 (-0.23; -0.07)	0.19 (0.15; 0.22)
Xanthurenic acid	-0.04 (-0.08; 0.00)	-0.26 (-0.34; -0.18)	0.07 (-0.02; 0.16)	-0.08 (-0.16; 0.01)	0.11 (0.07; 0.14)
Tryptophan	-0.09 (-0.13; -0.05)	-0.47 (-0.55; -0.40)	0.01 (-0.07; 0.10)	-0.02 (-0.10; 0.06)	0.09 (0.06; 0.12)
**Renal and endothelial functions**					
SDMA	0.31 (0.28; 0.35)	-0.26 (-0.33; -0.19)	0.01 (-0.07; 0.09)	-0.01 (-0.09; 0.07)	-0.09 (-0.12; -0.06)
ADMA	0.21 (0.18; 0.25)	-0.04 (-0.12; 0.03)	0.00 (-0.08; 0.09)	0.13 (0.05; 0.21)	0.08 (0.04; 0.11)
Creatinine	0.15 (0.11; 0.18)	-0.91 (-0.98; -0.84)	0.05 (-0.02; 0.13)	-0.06 (-0.14; 0.01)	0.02 (-0.01; 0.05)
Arginine	0.04 (0.01; 0.07)	-0.08 (-0.14; -0.02)	-0.03 (-0.10; 0.03)	0.03 (-0.03; 0.09)	-0.05 (-0.07; -0.02)
Homoarginine	-0.14 (-0.18; -0.11)	-0.41 (-0.49; -0.34)	0.01 (-0.07; 0.09)	-0.26 (-0.34; -0.18)	0.18 (0.15; 0.21)
